# *Hovenia dulcis* Thumberg: Phytochemistry, Pharmacology, Toxicology and Regulatory Framework for Its Use in the European Union

**DOI:** 10.3390/molecules26040903

**Published:** 2021-02-09

**Authors:** Gianluca Sferrazza, Gloria Brusotti, Manuela Zonfrillo, Caterina Temporini, Sara Tengattini, Monica Bononi, Fernando Tateo, Enrica Calleri, Pasquale Pierimarchi

**Affiliations:** 1 Institute of Translational Pharmacology, National Research Council, Via Fosso del Cavaliere 100, 00133 Rome, Italy; manuela.zonfrillo@ift.cnr.it (M.Z.); pasquale.pierimarchi@ift.cnr.it (P.P.); 2 Department of Drug Sciences, University of Pavia, Viale Taramelli 12, 27100 Pavia, Italy; caterina.temporini@unipv.it (C.T.); sara.tengattini@unipv.it (S.T.); or enrica.calleri@eufan.it (E.C.); 3 euFAN s.r.l, spin-off University of Pavia, Via Fratelli Cuzio 42, 27100 Pavia, Italy or monica.bononi@unimi.it (M.B.); or fernando.tateo@unimi.it (F.T.); 4 Department of Agricultural and Environmental Sciences, University of Milan, Via Celoria 2, 20133 Milan, Italy

**Keywords:** *Hovenia dulcis*, traditional medicine, phytochemistry, pharmacology, toxicology, regulatory science

## Abstract

*Hovenia dulcis* Thunberg is an herbal plant, belonging to the Rhamnaceae family, widespread in west Asia, USA, Australia and New Zealand, but still almost unknown in Western countries. H. *dulcis* has been described to possess several pharmacological properties, such as antidiabetic, anticancer, antioxidant, anti-inflammatory and hepatoprotective, especially in the hangover treatment, validating its use as an herbal remedy in the Chinese Traditional Medicine. These biological properties are related to a variety of secondary metabolites synthesized by the different plant parts. Root, bark and leaves are rich of dammarane-type triterpene saponins; dihydrokaempferol, quercetin, 3,3′,5′,5,7-pentahydroflavone and dihydromyricetin are flavonoids isolated from the seeds; fruits contain mainly dihydroflavonols, such as dihydromyricetin (or ampelopsin) and hovenodulinol, and flavonols such as myricetin and gallocatechin; alkaloids were found in root, barks (frangulanin) and seeds (perlolyrin), and organic acids (vanillic and ferulic) in hot water extract from seeds. Finally, peduncles have plenty of polysaccharides which justify the use as a food supplement. The aim of this work is to review the whole scientific production, with special focus on the last decade, in order to update phytochemistry, biological activities, nutritional properties, toxicological aspect and regulatory classification of *H. dulcis* extracts for its use in the European Union.

## 1. Introduction and *Hovenia dulcis* Traditional Uses

*Hovenia dulcis* Thunberg is an herbal plant belonging to the Rhamnaceae family. It is indigenous and widespread in East Asia, where is commonly known as Chinese Raisin Tree, Coral Tree, Japanese Raisin Tree, Korean Raisin Tree, Oriental Raisin Tree, while in USA, Australia, New Zealand and Central Africa, it has been introduced as an ornamental plant [1]. Among the Genus *Hovenia, H. dulcis Thunb., H. acerba Lindl*. and *H. dulcis var. tomentella* are known as herbal remedies in the ancient medicine; particularly, *H. dulcis* extracts are used in the Chinese Traditional Medicine in the treatment of several diseases. The whole plant extract is helpful in the hangover syndrome, decreasing alcohol concentration in blood, promoting clearing of alcohol and elimination of free radicals and avoiding dysfunction linked to alcohol abuse [1,2]. Fruits and peduncles possess antimicrobial, antioxidant and antidiabetic activities, while seeds can be used in the treatment of alcohol intoxication for their diuretic properties. In Japan, China and Korea, fruits are also used as ingredients for food supplements and nutraceuticals [3,4]. All these biological properties can be related to the variety of secondary metabolites synthesized by the different plant parts. In 2003, Xu et al. summarized the phytochemical composition of medicinal plants belonging to genus *Hovenia* [5], highlighting the presence of the following chemical families: triterpenoids, flavonoids, alkaloids, polysaccharides and organic acids. In particular, root, bark and leaves of *H. dulcis* are rich of dammarane-type triterpene saponins; dihydrokaempferol, quercetin, 3,3′,5′,5,7-pentahydroflavone and dihydromyricetin are flavonoids isolated from the seeds; fruits contain mainly dihydroflavonols such as dihydromyricetin (or ampelopsin) and hovenodulinol, and flavonols such as myricetin and gallocatechin; alkaloids were found in root, barks (frangulanin) and seeds (perlolyrin), and organic acids (vanillic and ferulic) in hot water extract from seeds. Finally, peduncles have plenty of polysaccharides [6]. Although *H. dulcis* seems to be a promising source of potential new drug and ingredient for food supplements, a literature survey using *Hovenia dulcis* as a keyword in SciFinder Scholar (1794 references), PubMed (76 references), Web of Science (188 references), Scopus (243 references) and Science Direct (218 references) as databases, highlighted a lack of scientific evidence. To date, SciFinder Scholar showed the highest number of published papers, 1794, with only 172 in English language, among which 114 were from the last decade; 9 reviews articles, 5 in English language, 3 in the last decade; 1522 patents, 19 in English language, 10 in the last decade. Thus, the aim of this work is to review the whole scientific production, with special attention to the last decade, in order to describe phytochemistry, biological activities and mechanism of action, and nutritional properties of *H. dulcis* extracts. Papers in their original language, rather than English, were not considered because they were not readable by the authors and thus unsuitable for critical analysis.

## 2. Phytochemistry

The first report on the chemical composition of *H. dulcis* dates back to 1973, when Takai et al. [7] described the isolation of the alkaloid frangulanin from its roots and bark. In 2003, Xu et al. [5] updated the list of bioactive compounds up to 2002, when Lee et al. [8] reported the isolation of hovenodulinol from the fruits. In almost 30 years, 54 compounds were isolated from fruits, seeds, roots and bark of *H. dulcis* and fully characterized. In 2005, Li et al. [9] reported the presence of (−) catechin, 2,3,4 trihydrobenzoic acid and (+) afzelechin in *H. dulcis* stem bark extract, known compounds but never found before in *H. dulcis* nor in the genus *Hovenia*. In 2012, Whang et al. [6] described for the first time the composition of the polysaccharides obtained from peduncles of *H. dulcis*: mainly Galattose and Arabinose and high contents of uronic acid, crucial for the immunostimulatory activity [10]. A novel kaempferol triglycoside was isolated from the leaves of *H dulcis*, together with 7 known compounds, never isolated before in this plant [11]; in 2017, six new triterpene esters were isolated from the roots [12]; finally, in 2018, the new compound 2-methoxybenzoic acid-5-*O*-α-L-rhamnopyranoside was isolated from branches of Korean *H. dulcis* [4]. From 2005 to now, a further 17 compounds have been isolated from different parts of *H. dulcis* and are summarized in Table 1.

### (+)-Dihydromyricetin (Ampelopsin)

Among all the secondary metabolites isolated from different parts of *H. dulcis,* (+)-Dihydromyricetin (DHM) or ampelopsin, discovered in seeds and fruits in 1997 [13,14], seems to be responsible for most of the biological properties claimed for this plant. DHM belongs to a chemical class of secondary metabolites, named flavonoids, secreted by several plants distributed worldwide and showing different biological properties. Ampelopsin was firstly isolated from *Ampelopsis meliaefolia* by Kotake and Kubota in 1940 [15]; in 1996, Zhou et al. [16] and Liu et al. [17] described the isolation of DHM from *Ampelopsis grossedentata* and its pharmacological activity on K^+^/Na^+^ channels [16] and the inhibitory effects of ampelopsin on tyrosinase [17], respectively. One year later, DHM was isolated for the first time from *H. dulcis* and described as the bioactive component responsible for the hepatoprotective activity claimed for this plant [13,14]. In the same year, 1997, a Japanese study described the inhibitory effect of *H. dulcis* methanol extract on the alcohol-induced muscular relaxation, highlighting DHM as the most bioactive compound [18]. In 2012, Shen et al. demonstrated the role of DHM in counteracting alcohol intoxication and dependence and suggested it as a therapeutic candidate in alcohol abuse syndrome [19]; in 2014, Zhou et al. [20] reported a study showing how DHM treatment can significantly lower the levels of blood glucose and insulin and in 2017, DHM was found to be responsible for the antiangiogenic activity ascribed to *H. dulcis* and suggested it as potential drug candidate for preventing angiogenesis-related disease, including cancer [21]. In 2018, Dong et al. proposed a mechanism of action concerning the hepatoprotective activity of *H. dulcis* fruit extract, and a further study of the same authors elucidated the direct involvement of DHM in the metabolism of acetaminophen endowing the use of fruits as an herbal remedy for preventing acetaminophen-induced liver injury [22,23]. The potential application of DHM as a drug or active component in food supplements is limited by its poor chemical stability and poor bioavailability [24]; however, in the last decade, new drug delivery systems have been proposed to overcome these disadvantages [25,26,27,28,29,30,31]. DHM is responsible for other pharmacological activities not related to *H. dulcis*, and thus that are not discussed in this paper [32].

## 3. Pharmacological Activities of *H. dulcis* Extracts

The biological activities of *H. dulcis* crude extracts and secondary metabolites isolated from them highlighted promising pharmacological effects in vitro and in vivo, the details of which are described below and summarized in Table 2.

### 3.1. Acute Alcohol Detoxification Effect and Anti-Hangover Activity

For a long time, *H. dulcis* has been utilized in Chinese and Korean traditional medicine to treat acute alcohol intoxication. This biological activity has been extensively studied, demonstrating that *H. Dulcis* extracts reduce alcohol concentration in blood [1]. Earlier studies in East Asia demonstrated that *H. Dulcis* extracts increase the activity of ADH and ALDH, contributing to reduce alcohol concentration after ingestion. A rapid in vitro screening performed by Xu et al. showed that *H. dulcis* fruits and stem extracts increase both ADH and ALDH activity measured through microplate reader assay [33]. This biological effect was also confirmed by another study that compared the detoxification activity of *H. dulcis* from China and Korea. The administration of crude seeds’ hot water extracts to CD^®^ (Sprague Dawley) rats, 30 min prior to alcohol ingestion, enhances the activity of ADH and ALDH more than control groups, reducing blood alcohol concentration. This effect was further enhanced when the crude extracts were partitioned with solvents. Interestingly, the partition of crude extracts improves the activity of ADH by 60% compared to the control. Contrarily, the ALDH activity was most affected by crude extracts. No significant differences in efficacy were observed between Korea and China *H. dulcis* extracts [34]. Similar results were obtained by two other in vivo experiments conducted by Okuma et al. [35] and Chen et al. [36] Both studies showed a decrease of blood alcohol and acetaldehyde levels in rats and mice [35]. Specifically, the administration of *H. dulcis* aqueous extract [36] significantly increased ADH activity in the liver of mice compared to the control group. The same results were obtained by Du et al. [37] when mice were treated orally with 60% ethanol (10 mL/kg) and *H. dulcis* semen extracts (150, 300, 600 mg/kg/day), for 4 consecutive days. The middle and higher dose of *H. dulcis* extracts significantly decreased the blood alcohol level at 300 and 600 mg/kg [37]. This evidence led Korean researchers to fill a patent application that covers technical aspects related to extraction and isolation of hovenodulinol from *H. dulcis* fruits and its bioactivity against alcohol toxicity [8]. The patent describes the isolation and identification of hovenodulinol from water and ethanol fruits extracts. A preliminary in vitro test on human hepatic cell line WRL-68 showed no toxic effect. The administration of 1 mg/kg of hovenodulinol to Sprague-Dawley rats, after alcohol ingestion, showed good properties in reducing blood alcohol and aldehyde concentration, from 30 min to 6 h, after alcohol ingestion, compared to the control group. The effect on ADH and ALDH activity in liver was also confirmed with an increasing enzymes activity of 42.6% and 59.8%, respectively. The enzymes activity in the control group was 28.3% for ADH and 29.1% for ALDH. Also, the GST activity was enhanced by 179% after the treatment with 1 mg/L of hovenodulinol. The authors demonstrated a similar effect when the test compound was administered to 20 healthy men. A reduction of alcohol and aldehyde was observed in saliva and exhaled breath in the treatment (5 mg/kg of hovenodulinol) versus control group [8]. Recently, a randomized controlled crossover trial evaluating the anti-hangover effect of freeze-dried aqueous extract of *H. dulcis* fruit was conducted [38]. Fruits were boiled with distilled water for 4 h, filtered, and concentrated. The extract was standardized with quercetin at 5.9–8.9 mg/g. Twenty-six eligible male adults were enrolled and allocated to placebo or treatment group with subsequent crossover. Hangover was induced with the administration of 360 mL of Korean Soju (50 g alcohol). In order to explore the mechanism underlying the anti-hangover effect, only subjects with heterozygous ALDH2 were included. Furthermore, blood alcohol, acetaldehyde and inflammatory cytokines were measured over time, evaluating the potential association with a score obtained through the administration of a questionnaire that evaluates hangover symptoms. The authors also evaluated the possible influence of CYP2E1 polymorphism on the relationships explored. The results demonstrated no difference between groups for blood alcohol and acetaldehyde concentrations, while a significant decrease in hangover symptom scores was observed in the treatment group compared to the placebo group. Significant differences between groups were also observed on IL-6, IL-10, IL-10/IL-6 ratio and AST levels. Cytokines level was positively correlated with total hangover symptom scores while the presence of CYP2E1 polymorphism can modify it. The authors concluded by stating that the pharmacological effect of *H. dulcis* extract on alcohol hangovers might be associated with the regulation of inflammatory response [38].

### 3.2. Hepatoprotective and Antifibrotic Activities

Several in vivo studies have been conducted to evaluate the hepatoprotective effect of *H. Dulcis* extract in various models of liver injuries induced by chemicals toxins [14,59,60].

#### 3.2.1. Effect on CCl_4_ Liver Injury

Hase et al. [14] conducted a bio-guided study of *H. dulcis* fruits extracts in order to evaluate the hepatoprotective effect of this plant. Male Sprague-Dawley rats were treated with 100 mg/kg of methanol or water *H. dulcis* fruits extracts twice daily for 1 week, before CCl_4_ administration. Only methanol extract showed a significant hepatoprotective effect compared to the CCl_4_-treated group. Serum AST and ALT levels, 24 h after CCl_4_ intoxication, were 933 ± 114 and 730 ± 212 U/L respectively, for water and methanol extract-treated groups. In the methanol-treated group, AST and ALT levels were significantly lower, 311 ± 94 and 175 ± 65 U/L, respectively. Methanol extract was further suspended in water and partitioned with EtOAc (ethyl acetate), obtaining EtOAc soluble and insoluble fractions. The hepatoprotective effect of these two fractions were evaluated in CCl_4_-induced rat hepatocyte injury in vitro. The EtOAc soluble fraction was more active than insoluble and methanol extract. Therefore, it was subjected to chromatographic separation in order to obtain 7 fractions; fraction 4, the most active, was further purified, allowing the isolation of 2 compounds, identified as Myricetin and DHM. Only DHM showed a hepatoprotective effect in the same in vitro model indicated above [14]. Similar results were obtained by Kim et al. in an in vivo model of acute liver injury induced by CCl_4_. The authors demonstrated a significant reduction in AST and ALT levels in rats treated with *H. dulcis* extract compared to the control group [59]. The same pharmacological activity was highlighted in a chronic in vivo model of hepatic fibrosis induced by CCl_4_ administered for 6 weeks in 48 male Sprague-Dawley rats. The potential mechanism of action of the of *H. dulcis* extract hepatoprotective effect was determined through the evaluation of mRNA expression of MMP-13 and TIMP-1 in hepatic tissue. Results obtained showed that the mRNA expression of TIMP-1 was statistically reduced by the plant extract, and this effect is correlated with the reversion of hepatic fibrosis in the experimental group [39,40]. A more detailed study on the anti-fibrotic effect of *H. dulcis* fruit extract in rats was conducted by Lee et al [60]. The administration of 4.0 mL/kg of methanol fruit extract, diluted in distilled water at a final concentration of 20%, five times a week (for 6 weeks), reduced ALT, AST and bilirubin levels and expression volume of collagen I and III, compared to the control group. Furthermore, the treatment reduced the expression and accumulation of collagen I and III in liver tissue. Pathological images confirmed that in rats treated with extract + CCl_4_, the progression of fibrosis was inhibited more than in rats treated with CCl_4_ alone. Moreover, the in vitro test demonstrated that methanol extract inhibited hepatic stellate cell proliferation at all the concentrations tested (from 6 to 180 mcg/mL), without cytotoxic effects on cell viability [60]. The same antifibrotic effect was also reported from four ceanothane-type and lupane-type triterpenoids isolated from *H. dulcis* roots methanol extract, with IC_50_ values in the range of 15–50 µM [12]. The hepatoprotective activity of *H. dulcis* fruit ethanol extract was also demonstrated in a chronic hepatitis model induced by CCl_4_ administration in mice [61]. Molecular and histopathological alteration in liver, induced by CCl_4_, were reduced in mice treated with 0.5 or 1 mg/kg of extract. Histological analysis demonstrated that *H. dulcis* attenuates fibrosis and necrosis, inhibits hepatic lipid peroxidation and induces a significant reduction of biochemical markers of hepatocellular necrosis and hepatic levels of malondialdehyde (MDA), compared to mice treated with CCl_4_ alone. At the molecular level, the effect of *H. dulcis* on mRNA expression of hepatic collagen (α1) (I) and collagen (α1) (III) was confirmed by RT-qPCR analysis. Finally, the treatment increases the expression of methionine adenosyltransferase 2a (MAT2A), an enzyme involved in hepatic regeneration [61].

#### 3.2.2. Effect on Alcohol-Induced Liver Injury

The protective effect of *H. dulcis* extract on alcohol toxicity was demonstrated both for semen and fruit extracts. The administration of semen extract, at the concentration of 150, 300 and 600 mg/kg/day for 4 days, exerts a hepatoprotective effect in mice with acute alcohol-induced liver injury, without inducing toxic side effects. In *H. dulcis*-treated mice, the levels of ALT and AST were significantly decreased in concomitance with an increased activity of ADH, SOD, GST and GSH that contributed to metabolize alcohol, rapidly. Furthermore, an acute toxicity test was conducted to assess safety and lethal dose of oral semen extract administration. A single dose, up to 22 g/kg, did not cause death or toxic effects during the 14 days of observation [37]. A study conducted by Wang et al. [6] focused the attention on the assessment of antioxidant activity of low molecular weight constituents of *H. dulcis* peduncles extract, characterized by the presence of polysaccharides. A first extraction with ethanol was performed in order to remove small molecules and oligosaccharides. The residues were then extracted with hot water followed by treatment with ethanol and macroporous resin. Galactose, arabinose, rhamnose and galacturonic acid were found to be the main components of the obtained fraction. Three different concentrations were administered: 100, 350 and 600 mg/kg, once daily for 20 days. Results demonstrated strong antioxidant activity properties both in vitro, due to high superoxide radical scavenging activity and significant inhibition effect on lipid peroxidation, and in vivo, restoring SOD and glutathione peroxidase (GSH-Px) activities in liver of mice injured by ethanol. Serum ALT and AST concentration and liver level of MDA were significantly lower than in mice treated with ethanol only. These results suggest that the hepatoprotective effect of *H. dulcis* peduncles extract is mediated via the antioxidant action. The polysaccharide fraction is one of the active principles contributing to the biological activities and traditional uses described for *H. dulcis* [6]. The same biological activity of polysaccharides fraction of *H. dulcis* was described and patented by Na et al. [62], where the hepatoprotective effect was demonstrated in an ex vivo model of liver toxicity induced by bromobenzene [60]. A study conducted by Yoshikawa et al. on *Hoveniae* semen seu fructus extracts reported an inhibitory action on the alcohol-induced muscular relaxation and a hepatoprotective effect on the d-galactosamine/lipopolysaccharide or CCl_4_-induced liver toxicity. Through the application of a bio-guided method, the authors reported DHM and hovenitin 1 as the molecules responsible for the biological activity on alcohol-induced muscular relaxation, but only hovenitin 1 demonstrated a hepatoprotective effect on liver toxicity induced by lipopolysaccharide (LPS) [18]. Cho et al. conducted a detailed study aimed to elucidate the molecular mechanisms supporting the hepatoprotective effect of *H. dulcis* extract on alcohol-induced liver toxicity [41]. *Hoveniae* semen seu fructus were extracted with hot water and three doses were selected (500, 250 and 125 mg/kg) and orally administered once a day, after 1 h of ethanol treatment for 14 days. The pharmacological effect of the extract was proved to exert statistically significant anti-inflammatory, anti-steatosis and antioxidant activities, at all dosages, in a dose-dependent manner. All molecular and histopathological markers related with alcohol intoxication were substantially improved by the experimental treatment compared to mice fed only with ethanol. Specifically, *H. dulcis* extract decreased AST, ALT, albumin, ALP, TG and γ-GTP levels in serum, and TG, TNF-α contents and CYP4502E1 activity in liver. The administration of *H. dulcis* extract enhanced hepatic GSH contents, SOD and CAT activities and modified mRNA expression of genes involved in hepatic lipogenic process, such as SREBP-1c, SCD1, ACC1, FAS, PPARγ and DGAT2, in fatty acid oxidation, including PPARα, ACO1 and CPT1, compared to the ethanol group. Oxidative stress and lipid peroxidation processes induced by ethanol were also normalized by the treatment through the decrease of immunoreactive hepatocytes cells positive to 4-hydroxynonenal and nitrotyrosine. Histopathological analysis revealed a significant and dose-dependent inhibition of steatosis, compared with the ethanol control mice. The molecular mechanism, underlying the hepatoprotective effect of *H. dulcis* extract in this model, is related to the strong antioxidant action and the regulation of genes involved in the lipogenic process and fatty acid oxidation in liver [41]. In a further model of liver hepatotoxicity, induced by chronic alcohol administration in rats, fruit water extract and seed ethanol extract of *H. dulcis* demonstrated anti-steatotic and anti-inflammatory effects at the concentrations of 300 and 500 mg/kg/day, respectively [63]. Both the extracts reduced hepatic and serum lipid contents and inflammatory markers, CRP, TNF-α and IL-6, compared to rats in the alcohol group. The decrease of hepatic fatty acid oxidative genes (Ppargc1a, Cpt1a and Acsl1) levels and the increase of myeloid differentiation primary response 88 (Myd88), TNF-α and CRP gene levels were positively regulated in the *H. dulcis* group. Both extracts also significantly reduce hepatic activities of fatty acid synthase and phosphatidate phosphohydrolase, plasma alcohol and acetaldehyde levels, hepatic enzyme activity and protein expression of CYP2E1, compared to the control group. This study provided further evidence that hepatoprotective action of *H. dulcis* extracts is exerted through the regulation of lipid and inflammation metabolism [63]. A further step to explain the therapeutic effect of *H. dulcis* in ethanol liver disease was recently provided by Ping et al. [64].

The authors explored the pharmacological effect on new molecular mechanisms characterizing the development of alcoholic liver disease. Recent findings attribute a central role to the gut–liver axis and its connection with microbiota. Ethanol assumption altered intestinal barrier function and gut microbiota, leading to an increase of endotoxin release, such as LPS, that promotes a critical crosstalk between liver and gut, exacerbating steatosis, inflammation and fibrosis in liver. LPS activates macrophage through activating the tool-like receptor 4 (TLR4) pathway and induces the release of NFkB and TNF-α. Sprague-Dawley rats were fed with a diet containing alcohol with or without semen *H. dulcis* water extract (300 and 600 mg/kg/d) for 8 weeks. The content of total flavonoid in semen was quantified and results showed that the extract contained 1.08% of DHM, 0.425% of dihydroquercetin and 1.4% of quercetin. Authors suggested that the therapeutic effect of the extract is due to the presence of these flavonoids, in accordance with literature data on their pharmacological effect on liver diseases. Results showed that the extract significantly ameliorated biochemical and histopathological markers such as ALT, AST, LDH and LPS. The increase of inflammatory molecules expression (TLR4, MyD88, NF-κB, Iκ-B, P-Iκ-B and TNF-α) in liver, induced by the activation of TLR4 receptor, were inhibited by experimental treatment compared to control group. Furthermore, the extract upregulated the expressions of zonula occludens-1 and occludin in the intestine, improved the barrier function and reduced the absorption of endotoxin, inhibiting the negative crosstalk induced by LPS between the gut and liver. For the first time, it was also demonstrated that *H. dulcis* modulates abnormalities of the gut–liver axis and regulates microbiota, promoting diversity and abundance of bacterial community and reducing microorganisms that release endotoxin into the enterohepatic circulation [64].

#### 3.2.3. Effect on Paracetamol-Induced Hepatotoxicity

A recent study conducted by Bui [65] demonstrated the acute hepatoprotective activity of *H. dulcis* extract on liver injuries induced by paracetamol in swiss albino mice. The experimental group was treated with 10 g/kg/day of *H. dulcis* ethanol fruit extract and paracetamol 400 mg/kg for a total of 8 days. Results demonstrated the hepatoprotective effect of the extract on paracetamol-induced toxicity through the reduction of AST, ALT, inflammation and necrosis compared to the control group [65]. In another study, conducted by Dong et al. [22] the hepatoprotective effect of ethanol fruit extract was evaluated on liver toxicity induced by paracetamol. Male C57BL/6 mice were allocated in different groups that included: control group, paracetamol group and experimental groups treated with 200, 400 or 800 mg/kg body weight, together with a single dose of paracetamol (300 mg/kg) to induce acute liver injury. The treatment was repeated every day, for five consecutive days. The pharmacological effect was evaluated by histopathological and biochemical analysis. Results demonstrated that *H. dulcis* extract reduced liver injury in a dose-dependent manner through multiple mechanisms of action. The extract reduced (a) the oxidative stress, increasing the expression of MDA, SOD and the concentration of GSH, (b) apoptosis of hepatocytes, decreasing cytochrome c, cleaved caspase-3 and caspase-9 expression, and increased B-cell lymphoma-2 (Bcl-2) expression, (c) the inhibition of CYP2E1, one of the enzymes that regulates the bioconversion of the drug that induces liver toxicity, and (d) serum marker enzymes ALT, AST and LDH. Finally, the extract also regulated bile acid and lipid homeostasis altered by paracetamol [22].

#### 3.2.4. Effect on lipopolysaccharide (LPS)/D-galactosamine (D-GalN)-Induced Liver Injury

The hepatoprotective effect of *H. dulcis* extract was also reported in immunological liver toxicity. Hase et al. [66] evaluated the pharmacological effect of *H. dulcis* water and methanol fruit extracts on LPS-induced liver injury in chronic ethanol-fed rats. A significant decrease of blood ALT and AST levels, accumulation of hepatic triglyceride, total cholesterol and malondialdehyde was demonstrated compared to the control group. Instead, no difference was observed between control and methanol extract-treated groups [66]. In another similar experimental study, however, the same authors reported that only methanol *H. dulcis* fruit extract exerts a hepatoprotective effect on LPS-induced rats’ liver toxicity. The results showed that treatment with methanol extract reduced ALT level by 75.9% compared to the control group. Furthermore, only 27.2% mice died in the methanol extract-treated group compared to 62.6% in the control group. No significant effect for both parameters was observed in the water extract-treated group [14]. Concerning the investigation of molecules contributing to the hepatoprotective action on immunological liver toxicity, Yoshikawa et al. [18] reported that the active compound was hovenitin I, a molecule isolated from *Hoveniae* semen seu fructus extracts.

### 3.3. Anticancer Activity

In vitro studies on the cytotoxic activity of *H. dulcis* extracts demonstrate antitumor properties in different cell lines. Castro et al. [42] reported a specific cytotoxicity, against P2/0 mouse myeloma and lymphoma cells, belonging to *H. dulcis* pseudo-fruits ethanol extract. Morales et al. [3] investigated the anticancer and hepatotoxicity activity of hydro-methanolic extracts of *H. dulcis* pseudo-fruits at different maturation stages. Cancer cell lines used in this study included: MCF-7 (breast carcinoma), NCI-H460 (non-small cell lung cancer), HeLa (cervical carcinoma), HepG2 (hepatocellular carcinoma) and HCT15 (colon carcinoma). Results demonstrated that only extracts obtained by immature pseudo-fruits showed antitumor properties and HCT15 and HepG2 were the most sensible cell lines (50% growth inhibition at 8.58 and 82.34 µg/mL, respectively). No hepatotoxicity on non-tumor liver primary culture (PLP2) was observed for all tested extracts [3]. Park et al. demonstrated that *H. dulcis* leaves’ methanol extract significantly inhibited the growth of HT29 and HepG2 cell lines. The maximum inhibition rate was of 80% at 100 μg/mL. Chloroform and hexane fractions, obtained from methanol extract, showed similar activity. No cytotoxicity effect was observed on the human liver cell line under the same conditions [43]. A recent study investigated the anticancer effect of *H. dulcis* branches ethanol extract and DHM, using in vitro and in vivo angiogenesis assays. Human umbilical vein endothelial cells (HUVECs) were used to evaluate cytotoxicity and a potential mechanism underlying the antiangiogenic action. The viability of HUVECs was not affected by the *H. dulcis* extract up to 100 μg/mL. Contrarily, the proliferation of HUVECs stimulated by vascular-endothelial growth factor (VEGF) was significant, in a dose-dependent manner, when treated with 25, 50 and 100 μg/mL of the extract. Moreover, *H. dulcis* ethanol extract inhibited invasiveness, tube-forming ability and migration of HUVECs induced by VEGF. The anti-angiogenic activity was also confirmed in vivo through the chorioallantoic membrane (CAM) assay, without inducing cytotoxicity. The analysis of the molecular pathway involved in this action revealed that *H. dulcis* ethanol extract inhibited VEGFR2-mediated downstream signaling cascades, reducing STAT3, AKT, ERK1/2 phosphorylation and the protein expression of VEGFR2, MMP-2, MMP-9 and cyclin D1 in HUVECs. In HepG2 cells, at the concentration of 100 μg/mL, the extract inhibited the expression of HIF-1α. DHM, one of the main secondary metabolites secreted by *H. dulcis*, showed the same antiangiogenic activity of the extract, through the inhibition of VEGFR2 signal transduction and suppressing the expression of HIF-1α/VEGF [21]; thus, in this case, the claimed biological activity was linked to a specific compound and not to the whole phytocomplex.

### 3.4. Antiallergic Activity

Four bioactive triterpene glycosides, Hovenidulciosides A_1_, A_2_, B_1_ and B_2_, were isolated from *Hovenia* semen seu fructus methanol extract. All compounds demonstrated inhibitory activity on the histamine release from rat mast cells induced by compound 48/80 and calcium ionophore A-23187 [44,45,46]. A recent study conducted by Lim et al. [47] investigated the anti-inflammatory and anti-allergic effect of three *H. dulcis* ethanol extracts obtained from fruits, branches and leaves. Antigen-stimulated mast cell-like cell line rat basophilic leukemia (RBL)-2H3 and a passive cutaneous anaphylaxis (PCA) mouse were chosen as in vitro and in vivo models. The RBL-2H3 cell line is a histamine-releasing cell line used in inflammation and immunological research. Cells were sensitized with dinitrophenol, then treated with the extract at the concentration of 5, 10 or 20 μg mL^−1^. Evaluation conducted at the molecular level revealed that *H. dulcis* branches extract inhibits inflammatory and allergic mediators in the RBL-2H3 cell line. Specifically, only *H. dulcis* branches extract showed anti-allergic activity both in vitro and in vivo. Results obtained in vitro demonstrated that the treatment induces the inhibition of β-hexosaminidase (that indicates the inhibition of degranulation) and histamine release. The production and expression of COX2, PGE2, interleukin-4, TNF-α and NFKB was also significantly inhibited. Furthermore, the authors investigated the effect of *H. dulcis* branches extract on FcεRI and MAPK, two molecular pathways known to be involved in inflammatory and allergic disease. The treatment with the extract was able to suppress the FcεRI pathway and the downstream signaling involved in mast cell activation and degranulation. Also, the inhibition of some components of the MAPK pathway, ERK and p38, involved in cytokine secretion, was proven. The anti-allergic activity of the extract was then investigated in a mast cell-dependent passive cutaneous anaphylaxis (PCA) mouse model induced by the administration of dinitrophenyl-IgE. The effect of *H. dulcis* branches extract, orally administered (100 or 300 mg kg^−1^), was compared to the antihistamine drug cetirizine (20 mg kg^−1^). Both treatments suppressed the antigen stimulated PCA response compared to the control group. The authors isolated 8 compounds from the extract, including: ferulic acid, vanillic acid, methyl vanillate, 2,3,4-trihydroxybenzoic acid, Taxifolin, Pinosylvin, 3,5-dihydrokaempferol and (−)-Gallotechin. They found that taxifolin, dihydro-kaempferol and pinosylvin inhibited β-hexosaminidase release in antigen-stimulated RBL-2H3 cells. Pinosylvin showed the most potent inhibitory effect. The mechanism of pinosylvin on IgE-sensitized RBL-2H3 cells was further investigated. No toxic effect on RBL-2H3 cell viability was observed at the concentration of 5–20 μg mL^−1^. Pinosylvin inhibited the release of proinflammatory mediators releasing IL-4, TNF-α and PGE2, and the expression of COX-2, IL-4, TNF-α, NFKB1 and NFKB2 in RBL-2H3 cells treated with IgE [47].

### 3.5. Anti-Inflammatory and Analgesic Activity

Different studies reported above highlighted the anti-inflammatory activity of *H. dulcis* extracts explicated through the inhibition of pathway and mediators known to be crucial for the development of inflammatory reaction, such as NFKB, TNF-α, IL-1, IL-10, etc. [48,49,67,68]. The anti-inflammatory effect of *H. dulcis* fruits ethanol extract in a mouse macrophage RAW 276.7 cells model was investigated. Results showed that *H. dulcis* extract significantly inhibited, in a dose-dependent manner, the expression of NO, nitric oxide synthase, COX-2, interleukin-1b, TNF-α, the nuclear translocation of NF-kB, p65 and the phosphorylation of IkB in the cytoplasm [67]. A similar in vitro study conducted by Jeong et al. [48] assessed the anti-inflammatory effect and the mechanism of action of *Hoveniae* semen seu fructus extracts in the murine macrophage cell line (RAW 264.7) and mouse primary macrophages. They demonstrated that *H. dulcis* ethanol extract (10, 30 and 50 mg/mL) strongly inhibited, in a concentration-dependent manner, the phosphorylation of MAPK and reduced the activation of activator protein-1 (AP-1), janus kinase-2 (JAK2)/STAT and NF-κB in LPS-stimulated RAW 264.7 cells. The expression of NO and iNOS, TNF-α, IL-6 and IL-1β was also significantly attenuated. In further analyses, DHM, taxifolin and myricetin were identified as the bioactive molecules responsible for the anti-inflammatory effect of the extract. In fact, each compound inhibited the inflammatory response in LPS-stimulated macrophages [48]. The anti-inflammatory action of *H. dulcis* extract was also confirmed in an in vivo model of atopic dermatitis-like skin lesions induced by 2,4-dinitrochlorobenzene (DNCB) in NC/Nga mice and TNF-α/interferon (IFN) gamma-induced chemokines production in spontaneously immortalized human keratinocytes (HaCaT) cell lines. The study design included 5 groups: control, DNCB, dexamethasone, *H. dulcis* branches (HDB) and extract (50 and 200 mg/kg plus DNCB). The extract and dexamethasone were oral administered once a day for 5 weeks. In vitro results showed that the extract significantly reduced MAPK signaling and cytokine production. The treatment also regulated the production of TARC (thymus and activation-regulated chemokine) and MDC (macrophage-derived chemokine) by HaCat cells stimulated by TNF-alfa and IFN-gamma. The anti-inflammatory effect of the extract’s main components (2,3,4-trihydroxybenzoic acid ferulic acid, vanillic acid, methyl vanillate, 3,5-dihydrokaempferol and pinosylvin) was evaluated in the same in vitro model. Methyl vanillate demonstrated the most potent anti-inflammatory action, inhibiting the expression of TNF-α, IL-6, TARC, MDC, ERK, c-jun N-terminal kinase and p38MAPK. In vivo data showed that HDB extract reduced epidermal thickness and dermal infiltration of cytokine-expressing inflammatory cells. Moreover, serum IgE and IgG2a levels and the expression of mRNA for Th1 and Th2-related mediators in skin lesions were significantly reduced. The authors concluded the study, suggesting that the mechanism of action of HDB in an atopic dermatitis model is mediated by the regulation of Th1 and Th2 responses and consequent expression of inflammatory mediators [49]. Another in vivo study conducted by Lee et al. [60] investigated the analgesic effect of *H. dulcis* extract on an inflammatory orofacial pain model induced by formalin. Rats were divided in four groups: (a) control, (b) 5% formalin, (c) 5% formalin + 4.5 mL/kg of *H. dulcis* extract and (d) 5% formalin + distilled water administration. Results obtained from the study reported a significant reduction of orofacial pain in *H. dulcis*-treated animals compared to other groups. Western blot analyses, conducted on markers involved in pain regulation and inflammation, revealed that the extract inhibited p38MAPK, iNOS and Nrf2 in the brain.

### 3.6. Laxative Activity

Evidence of laxative activity of *H. dulcis* extracts were derived from two recent in vivo studies. In the first study, Choi et al. [69] described the laxative properties of *H. dulcis* branches after extraction with water followed by partition in hexane, chloroform, ethyl acetate and butanol. The laxative effects were assessed on low-fiber diet-induced constipation in Sprague-Dawley rats by measuring spasmogenic activity and intestinal transit of charcoal meal and stool parameters. Results showed that only water extract (100 and 200 mg/kg) had a positive effect, improving the intestinal transit of charcoal meal and the frequency and weight of stools. Moreover, the contractile responses of isolated rat colon were significantly enhanced by the water extract. Finally, the authors identified vanillic acid as the active molecule of the extract with spasmogenic activity on an isolated rat colon test. The second in vivo study evaluated the laxative activity of hot-water extracts of *H. dulcis,* in two chronic constipation models: loperamide-induced constipation and low-fiber diet-induced constipation. Results showed an increase of stool parameters (fecal number, weight and water content), and intestinal transit compared to loperamide and low-fiber diet groups [50].

### 3.7. Antimicrobial Activity

The antimicrobial effect of *H. dulcis* was proven against Gram-positive bacteria, Gram-negative bacteria, parasites and yeasts [1]. A study conducted on methanol-soluble fraction of *H. dulcis* hot-water extracts lead to the isolation of vanillic and ferulic acids as active molecules with antimicrobial activity against Gram-positive bacteria (*Staphylococcus aureus*, *Staphylococcus epidermidis, Bacillus subtilis, Micrococcus luteus, Streptococcus mutans, Lactobacillus plantarum, Lactobacillus brevis, Leuconostoc mesenteroides* and *Pediococcus damnosus),* Gram-negative bacteria (*Escherichia coli, Pseudomonas aeruginosa* and *Salmonella typhi*) and yeast (*Candida albicans*) [51]. Cho et al. isolated 3(Z)-dodecenedioic acid from *H. dulcis* leaves and demonstrated its antimicrobial activity against *Staphylococcus aureus* and *Escherichia coli* [70]. The antimicrobial effect was also demonstrated for *H. dulcis* pseudo-fruits extracts [71]. A recent study conducted by Morales et al. reported that the antimicrobial activity of. *H. dulcis* pseudo-fruits extract is strictly correlated to the fruit development process. Extract of fruits in the immature stages showed high activity and low minimal inhibitory concentration values on *Staphylococcus epidermidis, Sthaphylococcus aureus* and *Pseudomonas aeruginosa*. The authors reported that the antimicrobial effect could be related to the flavonoids content. In fact, immature pseudo-fruits showed high content of catechin and quercetin derivatives [3]. The inhibitory activity against parasites was described by Gadelha et al. [72] and Castro et al. [42], demonstrating the ability of *H. dulcis* extracts to inhibit the growth of Giardia lamblia trophozoites (dichloromethane fraction from the methanol extract of leaves) and *Trypanosoma cruzi*, respectively.

### 3.8. Antidiabetic Activity

Both in vitro and in vivo studies have demonstrated the antidiabetic effect of *H. dulcis* extracts. Different in vitro studies reported the inhibitory effect of crude extracts against α-amylase and α-glucosidase [52,73,74,75]. Meng et al. [52] showed that total flavonoids, myricetin and quercetin obtained from *H. dulcis* ethanol seeds extract have an inhibitory effect on α-amylase and α-glucosidase. The IC_50_ related to the inhibitory activity of total flavonoids, myricetin and quercetin against that of α-amylase was 32.8, 662 and 770 μg mL^−1^, respectively. In the case of α-glucosidase, all three samples demonstrated a more potent inhibition with IC_50_ values of 8, 3 and 32 μg mL^−1^. The authors also demonstrated that the inhibition was reversible and competitive on α-amylase, while the effects on α-glucosidase were non-competitive. It was also reported that myricetin was the most promising compound since its activity was more effective than acarbose [52]. The in vitro inhibitory activity against α-amylase and α-glucosidase enzymes was also demonstrated for *H. dulcis* extract, mainly composed of polysaccharides, and their bioactivity was affected by the extraction method [73,74,75]. The antidiabetic effect was investigated and proven also on different in vivo models of diabetic mice and rats [53,76,77,78]. In an alloxan-induced diabetic model, the treatment of mice with glibenclamide or *H. dulcis* extract significantly lowered blood sugar levels and increased hepatic glycogen, compared to the control group [76]. The antidiabetic activity was also observed in streptozotocin-induced hyperglycemic mice and rats. Kim et al. demonstrated that the administration of *H. dulcis* peduncles water extract for 6 weeks at the concentration of 0.01 and 0.04 g/kg reduced blood glucose concentration and partially recovered pancreatic islets and pancreatic β-cells from the damage induced by streptozotocin. The authors correlated these effects with the antioxidant activity of the extract [53]. The same effect was shown in the study published by Lee et al. [60]. The treatment of streptozotocin-induced diabetic rats with 20 or 50 mg/kg^−1^ of ethyl acetate fraction from an 80% methanolic extract of *H. dulcis* fruits induced a decrease of plasma glucose, lipid peroxide, total cholesterol and triglycerides in liver microsomes and an increase of glutathione levels in the liver cytosol [77,78]. Furthermore, in a recent in silico analysis performed in order to identify potential pharmacological target of *H. dulcis* compounds (especially flavonoids) among the main pathways involved in diabetes mellitus, including insulin resistance, glucose level and chronic inflammation, it was suggested that *H. dulcis* flavonoids may exert their antidiabetic and anti-inflammatory activity through the modulation of AKT1 and glycogen synthase kinase 3 beta (GSK3β) [79].

### 3.9. Anti-Dyslipidemic and Antiadipogenic Activities

The anti-dyslipidemic and antiadipogenic activities of *H. dulcis* extracts were examined in a limited number of in vitro and in vivo models. Kim et al. assessed the antiadipogenic effect of *H. dulcis* fruit or stem water extracts in the 3T3-L1 preadipocytes cell line. The fruit extract (100 μg/mL^−1^) showed a significant dose-dependent inhibition of lipid accumulation, downregulating the expression of the PPARγ, CCAAT/enhancer-binding protein-α, adipocyte fatty acid-binding protein 2, adiponectin and resistin, with an inhibition rate of 29.33%, 54.36%, 34.5%, 55.69% and 60.39%, respectively. Moreover, the extract increases the phosphorylation of AMPK-α, liver kinase B1 and acetyl-CoA carboxylase [54]. In vivo studies also demonstrated the ability of *H. dulcis* extract to improve lipid metabolism. Pinto et al. [55] evaluated the effect of hydroalcoholic extract of *H. dulcis* fruit (50.0 and 100.0 mg/kg) and DHM (25.0 and 50.0 mg/kg) in hypercholesterolemic rats. The results demonstrated that both treatments significantly reduced total cholesterol and LDL-C, compared to the control. However, an increase of triglycerides and hepatic transaminases was observed only in rats treated with DHM, suggesting that the crude extract could be more useful than the compound alone [55]. The effect on lipid metabolism was also demonstrated by administering insoluble dietary fiber obtained from *H. dulcis* pomace and modified by ball milling and complex enzyme treatment. The treatment of mice with insoluble dietary fiber significantly slowed weight gain in hyperlipidemic mice, improved lipid metabolism (serum high-density lipoprotein cholesterol, total cholesterol, triglyceride and low-density lipoprotein cholesterol levels) and atherosclerosis and liver index, compared to the control group [74].

### 3.10. Antioxidant Activity

The antioxidant action of *H. dulcis* extracts has been demonstrated in numerous studies and this effect has been considered also as one of the mechanisms supporting the hepatoprotective and alcohol detoxification effects observed in various in vitro and in vivo studies [6,80]. A study conducted by Morales et al. [3] explored the antioxidant capacity of hydromethanolic extracts of *H. dulcis* pseudo-fruits through three in vitro assays: 2-2-diphenyl-1-picrylhdrazyl (DPPH) scavenging activity, reducing power (Ferricyanide/Prussian blue assay) and β-carotene/linoleate assay, in order to determine the inhibition of the lipid peroxidation process. The authors revealed that antioxidant effect was strongly correlated with the maturation process of pseudo-fruits. In fact, pseudo-fruits during the immature stage have higher antioxidant activity compared to those at the mature stage. This difference was explained due to the high presence of phenolic compounds during the immature stage. Similar results were observed when the antioxidant activity was evaluated through other antioxidant assays [3]. Other authors also confirmed the antioxidant activity of other botanical parts of *H. dulcis,* such as leaves and stem extracts [9,11]. Other studies exploring the antioxidant effect of the polysaccharide compounds and polyphenolic–protein–polysaccharide complexes extracted from peduncles or other parts reported a strong activity of these fractions, suggesting their applications in the functional food and medicine industries [6,56,57,81].

### 3.11. Anti-Osteoporotic Effect

An in vitro, ex vivo and in vivo study investigates the use of *H. dulcis* extract as potential treatment for osteoporosis [58]. Top-flash screening assay conducted on 350 plants indicated that *H. dulcis* water extract was an activator of Wnt/β-catenin signaling, one of the potential pharmacological targets for the development of anti-osteoporotic drugs. The activation of Wnt/β-catenin signaling promotes osteoblast differentiation, subsequent bone formation and suppresses osteoclastogenesis. *H. dulcis* water extract promotes bone formation in an ex vivo calvaria assay and activates Wnt/β-catenin signaling in a dose-dependent manner at the concentration of 5 and 50 μg/mL, without significant toxicity. Moreover, in primary calvarial osteoblasts treated with *H. dulcis* extract, the mRNA levels of the osteoblast differentiation markers such as RUNX2, BMP2, ALP and OCN were significantly increased. The expression of RANKL and OPG was reduced and increased, respectively. Hematoxylin and eosin staining showed a reduced calvaria thickness in a sample treated with the extract. The administration of *H. dulcis* extract (200 mg/kg for 4 weeks) in ICR mice induced an increase of femoral bone mass and thickness compared to in the control group. This activity was related to the activation of the Wnt/β-catenin pathway in trabecular and cortical bones of femurs. In order to identify the molecules activating Wnt/β-catenin, the authors tested 8 compounds in the same experiments, known to be part of *H. dulcis* extracts: vanillic acid, methyl vanillate, ferulic acid, myricetin, taxifolin, 2,3,4-trihydrobenzoic acid, dihydrokaempferol and gallocatechin. Methyl vanillate (20 uM) showed higher activity in all tests described above, demonstrating to induce osteogenesis promoting the expression of differentiation markers in a dose-dependent manner through the activation of the Wnt pathway. In fact, siRNA-mediated β-catenin knockdown suppressed the activation-regulated mediated by methyl vanillate. Further investigation confirmed the anabolic and anti-osteoporotic effect, reversing bone loss in ovariectomized mice through the increase of β-catenin expression in femoral trabecular and cortical bones. The effect was dose-dependent and did not induce toxicity [58].

### 3.12. Immunomodulatory Activity

Only one study, among those available in the literature, demonstrated the immunostimulatory activity of *H. dulcis* aqueous ethanol peduncle extract [10]. Wang et al. fractioned the extract and obtained three fractions (HDPS-1, HDPS-2 and HDPS-3) that were mainly composed by rhamnose, arabinose, galactose and galacturonic acid. However, HDPS-3 contained a higher content of galacturonic acid (40.5%) than HDPS-1 and HDPS-2 (1.8% and 7.6% respectively). Moreover, HDPS-1 had a much higher molecular weight than HDPS-2 or HDPS-3. In vitro studies demonstrated that all three fractions had immunostimulatory activities, stimulating the proliferation of splenocytes and activating peritoneal macrophages, enhancing phagocytosis, NO production and acid phosphatase activity. HDPS-1 was the most active fraction. The authors suggested that molecular weight, monosaccharide composition and uronic acid content were crucial for the immunostimulatory activity [10].

### 3.13. Neuroprotective Effect

A neuroprotective action of *H. dulcis* stem bark extract was reported by Li et al. [9]. Several fractions extracted from *H. dulcis* stem bark were assessed against glutamate-induced neurotoxicity in mouse hippocampal HT22 cells. EtOAc-soluble fraction from methanolic extract exhibited in vitro neuroprotective activity at the concentration of 5 μg/mL, increasing HT22 cell viability (71.3% ± 8.1%) compared to those treated with glutamate only (38.3% ± 4.1%). The fraction also possesses antioxidant activity against DPPH, ABTS and superoxide radical scavenging assay. A bioassay-guided method was applied leading to the identification of active compounds: (−)-catechin and (+)-afzelechin. Both molecules demonstrated neuroprotective and antioxidant activities [9].

## 4. Preclinical Pharmacokinetic and Herbal–Drug Interaction Study

### 4.1. Preclinical PK Study

To the best of our knowledge, the literature lacks in pharmacokinetic (PK) studies on *H. dulcis* extracts. Only one recent study explored the PK profile of active molecules known to be part of *H. dulcis* extracts. Yang et al. conducted a comparative pharmacokinetic study between dihydromyricetin, dihydroquercetin, myricetin and quercetin after oral administration of *Hoveniae* semen ethanol extract (HSEE) and mixture composed of the same four flavonoids (MF) [82]. The content of dihydromyricetin, dihydroquercetin, myricetin and quercetin in the HSEE was 299, 124, 80.0 and 23.4 mg/g, respectively. The PK study was conducted on eighteen male Sprague-Dawley rats randomly allocated in three groups: control group and those who orally received a single dose of HSEE or MF. The dose of HSEE administered via oral gavage was 0.333 g/kg, which corresponded to 100, 41, 27 and 7.8 mg/kg of dihydromyricetin, dihydroquercetin, myricetin and quercetin, respectively. The concentrations of the four flavonoids in MF solution were equal to the HSEE solution. The authors developed and validated a liquid chromatography-tandem mass spectrometry (LC-MS/MS) method for simultaneous detection of the 4 flavonoids in rat plasma. Results obtained suggested that all four flavonoids were rapidly absorbed, but the time of maximum concentration observed (T_max_) of quercetin in HSEE was significantly shorter than in MF. The authors suggested that this is probably due to other molecules characterizing HSEE, such as flavonoids (kaempferol, apigenin) and phenolic acids (vanillic acid, ferulic acid) that can alter the pH in the stomach, promoting the absorption rate and maximum concentration observed (C_max_) of quercetin. Furthermore, C_max_ and area under curves (AUC_0−t_ and AUC_0−∞_) of dihydromyricetin in HSEE were significantly higher than in MF. Finally, the mean elimination half-life (t_1/2_) of the four flavonoids in HSEE were rather longer than that in MF. The mean residence time (MRT) of the four compounds in HSEE was longer than in MF. The main statistical difference was observed in pharmacokinetic parameters of dihydroquercetin. In fact, relevant differences were observed for all parameters, such as AUC_0−t_, AUC_0−∞_, half-life (t_1/2_), mean residence time (MRT_0−t_), MRT_0−∞_ and volume of distribution during the terminal phase (Vz) of dihydroquercetin after administration of HSEE or MF. Scientific evidence obtained from this study could be crucial for the development of new formulation and pharmaceutical combination in order to improve the bioavailability of flavonoids and optimize the clinical use. Scientific findings obtained from the study are crucial in order to propose a new strategy for improving the ADME (absorption, distribution, metabolism, excretion) profile of dihydromyricetin and dihydroquercetin, two of the main active molecules of *H. dulcis* extract that have raised interest for their potential therapeutic application. The improvement of T_max_ and C_max_ and AUC values are probably induced by other known compounds of HSEE (such as vanillic acid, ferulic acid, kaempferol and apigenic) that can promote the absorption of dihydromyricetin and dihydroquercetin in the gastrointestinal system or inhibit their metabolism and excretion in kidney, thus improving their bioavailability [82,83]. Scientific data obtained from this study are crucial if we consider that a previous PK study on rats demonstrated that DHM is rarely absorbed in the gastrointestinal tract and has poor oral bioavailability [84,85].

### 4.2. In Vitro Herbal–Drug Interaction

Potential *H. dulcis*–drug interactions were investigated by Park et al. [83] through the evaluation of the inhibition effect of *H. dulcis* fruit water extract on the cytochrome P450 CYP enzyme activity using a human liver microsomes model. The *H. dulcis* fruit water extract was tested at different concentrations (1, 3, 10, 30 and 100 μg/mL) with and without a preincubation procedure. The validity of the bioassay was assessed with selective inhibitors of CYP isozymes: furafylline (CYP1A2), methoxsalen (CYP2A6), quercetin (CYP2C8), sulfaphenazole (CYP2C9), ticlopidine (CYP2C19), quinidine (CYP2D6) and ketoconazole (CYP3A4). Results obtained from the study showed that water extract of *H. dulcis* fruits did not cause relevant inhibition of CYP enzymes (IC_50_ values > 100 μg/mL) [86]. However, it has been reported in the literature and in some in vivo studies described above, that *H. dulcis* extracts, DHM and taxifolin alone have inhibitory effects on CYP enzymes [86,87]. This discrepancy among different studies may be related to the type of extract used and the effective content of DHM and taxifolin. It is also possible to suppose that other compounds of *H. dulcis* extract, besides DHM and taxifolin, could compensate their inhibitory effect on CYP enzymes [86,87].

## 5. Nutritional Properties

*H. dulcis* has a long history as a traditional medicine and functional food in East Asia. The characterization works on the edible fraction of *H. dulcis* derive from only recent experiences. The edible pseudo-fruit of *H. dulcis* is identified by the ripe peduncle and presents a flavor reminiscent of the pear, with good acceptability for human consumption [88]. The ripe peduncle is used for the preparation of juice, fermented juice, acetified derivative and for nutritional enrichment of baked products. The pulpy pseudo-fruit is commonly known as “Japanese grape.” A useful note to the initial knowledge of this pseudo-fruit is that of Maieves et al. [88], which considered sugar and polysaccharide fraction at different ripening stages of *H. dulcis*. As predictable, soluble sugars content increase during ripening, probably due to the hydrolysis of the starchy fraction and to the reduction of water content. Dietary fiber also spontaneously increases during the development of these pseudo-fruits, as well as the mineral content among which copper, calcium and manganese predominate. The performed verifications concerned soluble sugars, soluble and insoluble dietary fiber, vitamin C (such as ascorbic acid) and total carbohydrates. At the maximum ripening stage, total carbohydrates reach approximately 26%, with a percentage of humidity at 51%, the maximum content of vitamin C reaches 6–8%, and it is interesting to note that the value of the total fiber reaches 25% at full ripeness. The caloric intake increases from 15 (unripe stage) to 160 kcal (ripe stage) per 100 g fresh weight.

A work by Yang et al. [56] examines the attempt to solve the problem of low extraction yield of polysaccharide molecules. These authors adopt single-frequency (SF), double-frequency (DF) and triple-frequency (TF) ultrasonic technologies, and the maximum yield in polysaccharides is obtained through DF, at 58 °C in 33 min and at 28 and 40 kHz. The composition of the polysaccharides is studied by means of high-performance gel permeation chromatography technique, and the heteropolysaccharides result poly-dispersed in a wide array of molecular weights. Concerning the uronic acid content, the DF extraction produces the greatest amount of it. In rheological tests, polysaccharides have shown to possess excellent colloidal thickening and gelatinizing properties. These characteristics come out in favor of a product that can be widely used for various food purposes. Indeed, DF appears to be the best method for obtaining an extract with higher antioxidant properties. A strong chelating ability of iron is demonstrated in concentrated solution (4 mg/mL), increasingly evident in the extract produced through DF. In conclusion, the authors highlight the many potential applications in functional products for the industry. On polysaccharides, Liu et al. [89] employ alkaline extraction by stating that this extraction method is preferable for efficiency to the others, including ultrasonic extraction. This technique allowed the authors to isolate a different heteropolysaccharide denominated spent *H. dulcis* peduncles (SHDP). This polysaccharide has been characterized concerning the simple constituent sugars and their quantitative relationship. According to the authors, the heteropolysaccharide SHDP is able to increase the alcohol-dehydrogenase activity. A recent work by De Biaggi et al. [90] focuses on the importance of the content in catechins, reaching 148 mg/100 g fresh weight (gFW) of catechin and 8 mg/100 gFW of epicatechin. The content in tannins (62 mg/100 gFW of castalagin and vescalagin) is also detectable. Quinic acid is the most represented among organic acids (approximately 43 mg/100 gFW), while ascorbic and dehydroascorbic acid reach in total 44 mg/100 gFW. In conclusion, this work highlights, like other preceding ones, the content in tannins and catechins, and therefore the *H. dulcis* can be considered interesting material for research on its biological activities, taking into account the variations due to genotypes. Meng’s work [52] is aimed instead at the evaluation of total flavonoids, myricetin and quercetin. Regarding the inhibitory effects on α-amylase and α-glucosidase, the authors draw the conclusion that the flavonoids of *H. dulcis* allow to manage postprandial hyper-glycemia and consider this source of myricetin interesting for the nutraceutical industry.

## 6. Toxicity

As reported in the introduction, *H. dulcis* has been used as a traditional medicine and food supplement in East Asia for a long time. It is considered generally safe when used properly and in line with a specific therapeutic scheme. However, scientific evidence described in the literature about the safety of *H. dulcis* are mainly restricted to in vitro studies, and only few in vivo toxicity studies have been conducted.

### 6.1. In Vitro and In Vivo Studies

Several in vitro studies showed the absence of cytotoxicity of *H. dulcis* extracts against different cell lines. Morales et al. reported no hepatotoxicity on porcine liver cells (PLP2) of *H. dulcis* Thunb. pseudo-fruits hydromethanolic extracts at five different maturity stages. The GI_50_ (growth inhibitory 50%) values were >400 µg/mL for all the extracts tested [3]. Mouse calvarial osteoblasts treated for 72 h with 50 µg/mL of *H. dulcis* extract or 20 μM of methyl vanillate (one of the components of the extract) did not induce cytotoxicity. The administration of the extract and methyl vanillate (200 and 100 mg/kg respectively, for 5 sequential days each week for 4 weeks) did not cause weight changes or abnormality in liver tissue [58].

Three different studies investigated the effect of *H. dulcis* extracts on the viability on murine macrophages line RAW 264.7. Hu et al. reported that butanol, water and methanol extracts did not have cytotoxic effects at the concentration of 1 mg/mL, whereas ethyl acetate and chloroform extracts, at the same concentration, showed a marked cytotoxicity on RAW 264.7 cells, reducing its viability by 80% compared to the control [91]. In the study conducted by Park et al., ethanol extract of *H. dulcis* fruit did not cause cytotoxicity up to 100 µg/mL, whereas at the highest concentration tested (120 µg/mL), the extract produced a 25% reduction of macrophages’ viability [67]. Also, Jeong et al. demonstrated that ethanol *Hoveniae* semen seu fructus extract did not affect the RAW 264.7 viability, up to 50 μg/mL after 48 h of treatment [48]. Furthermore, no cytotoxicity was observed on rat basophilic leukemia RBL-2H3 cells when treated with branches, leaf and fruits extracts (5–20 μg/mL^−1^). In another study, it was demonstrated that branches extract did not induce toxicity below 10 μg/mL on HaCat cells [49]. Han et al. showed that the viability of human umbilical vein endothelial cells (HUVECs) was not affected when exposed to ethanol *H. dulcis* branches extract in a concentration range of 0–100 μg/mL for 72 h. A 50% reduction of cell viability was observed at 200 μg/mL [21]. The cytotoxicity of fruits extract of *H. dulcis* on 3T3-L1 cells was assessed at various concentrations (10–300 μg/mL^−1^). No significant toxicity was observed in the range of concentration of 10–100 μg/mL^−1^. A reduction of 25% of cell viability was reported at 300 μg/mL^−1^ [54]. The absence of cytotoxicity was reported also when hepatic stellate cells were treated with methanol extract of *H. dulcis* fruits (from 6 to 180 μg/mL) [60]. Same results were reported on HepG2 cells, which are hepatocyte-derived cells, when treated with *Hoveniae* semen cum fructus or seed extracts. No significant toxicity was reported up to 1000 μg/mL [80,92]. A single-dose acute toxicity study was conducted in mice that received *H. dulcis* semen extract from 1 to 22 g/kg. The results demonstrated that the administration of the extract was safe and did not result in any death or toxic effects during 14 days’ observation [36].

### 6.2. Human and Animal Toxicity

Despite the good tolerability and safety profile demonstrated by the literature, three cases of toxic hepatitis have been reported in Korea, among which, two in adult patients and one in a pediatric subject [93,94,95,96]. It has been indicated that the toxicity was probably due to a continuous misuse of *H. dulcis*. Concerning the pediatric clinical case, Kim et al. reported that a 3-year-old boy, after consuming *H. dulcis* water decoction every day for 1 year, developed a toxic hepatitis which evolved into hepatic failure requiring liver transplantation. The authors supposed that the toxicity was mainly based on idiosyncratic reactions [94]. Also, some cases of spontaneous poisoning in cattle and goats caused by *H. dulcis* fruits have been described. Animals developed an acute toxicity that led to their death [97,98]. Therefore, it is very crucial to carry out further research on safety profile of *H. dulcis* extracts in order to improve the correct use and discourage its misuse.

## 7. Regulatory Framework for the Use of *H. dulcis* Extract in Europe

*H. dulcis* has a long history as a traditional medicine and functional food in East Asia. It is estimated that 5.2% of Korean adults use *H. dulcis* food supplements. In fact, several products (tablets, capsule, tea drinks and beverage) based on *H. dulcis* extract are approved for food use and marketed in South Korea [99]. However, to date, not one product is available in the European market. The high number of bioactive components contained in hydrophilic extracts, such as flavonoids, polyphenols and polysaccharides, suggested a promising application of *H. dulcis*, not only in the pharmaceutical sector but also in the functional food field [89]. In Europe, new food ingredient materials can be authorized only after the evaluation of their safety, according to the novel food regulation. The introduction of novel food in the European market is regulated by the European Parliament and the Council on the basis of the Regulation (EU) 2015/2283 on novel foods that replaces Regulation (EC) No 258/97 and Regulation (EC) No 1852/2001. The new legislative framework introduced a centralized assessment and authorization procedure. The European Commission has the responsibility to authorize novel foods, establishing the conditions of use (of the food or the food ingredient) and its specification and labelling requirements. The European Food Safety Agency (EFSA) provides a scientific assessment in order to establish the safety profile. As reported by the European Commission, a novel food is “a food that had not been consumed to a significant degree by humans in the European Union (EU) before 15 May 1997, when the first Regulation on novel food came into force. Novel Food can be newly developed, innovative food, food produced using new technologies and production processes, as well as food which is or has been traditionally eaten outside of the EU” [100]. Briefly, the introduction of a novel food into the European market is based on the presentation of a dossier describing all data related to the history of use and its source, the composition and physicochemical properties, the production process, the nutritional aspects, the ADME (absorption, distribution, metabolism and excretion) profile, the safe use in humans based on toxicological and allergenic studies and the proposed uses and use levels [101]. The authorization procedure of a traditional food is based on the demonstration of the safety profile of its use in at least one country outside the EU for a period of at least 25 years. The EFSA evaluates the scientific aspects while the EU risk managers decide if the food might be considered a traditional food. The dossier should describe the identity of the traditional food, its origin, the composition, physicochemical properties, production process, stability data and evidence derived from its continued use in a third country. The safe use derived from a literature study on human use should be demonstrated and related to the proposed conditions of use for the EU [102]. According to the European Commission website, only one application for the authorization of dried fruit and peduncle extract of *H. dulcis* has been submitted [103]. The authorization was not granted due to an insufficient characterization of the quality and safety aspects, as reported by German, United Kingdom competent authorities [104] and the EFSA [99].

## 8. Conclusion and Future Perspectives

Although *H. Dulcis* extracts are well-known and used in the Chinese Traditional Medicine for the treatment of several diseases and as a food supplement in Japan, China and Korea, little is known about it in Western countries, so far. However, in the recent years, *H. dulcis* gained growing interest among the scientific community, due to the variety of biological activities, such as antidiabetic, anticancer, antioxidant, anti-inflammatory, hepatoprotective effects and in the hangover treatment, especially.

Secondary metabolites responsible for interesting biological activities are usually secreted from aerial or from non-aerial parts of a plant, unlikely from both of them. On the contrary, in *H. dulcis,* almost all the plant parts, such as root, bark, leaves, seeds, fruits and pseudo-fruits, are able to produce biologically active extracts. Triterpenoids, flavonoids, alkaloids, polysaccharides, organic acids, saponins, dihydroflavonols and flavonols are the well-known chemical families represented in *H. dulcis* and responsible for the claimed biological activities.

DHM, a dihydroflavonol belonging to the flavonoids family, resulted to be crucial for most of the pharmacological activities since it is secreted from different plant parts such as fruit, branches, semen seu fructus and seeds. Due to its role in counteracting alcohol intoxication, DHM seems to be a good candidate in alcohol abuse syndrome, but as described, is also involved in other different pharmacological activities.

Hovenodulinol is another dihydroflavonol found to be helpful in the hangover syndrome only, and is secreted from fruit, semen and stem bark.

Pseudo-fruits represent an interesting part of the plant since their activity is correlated with the maturation stage. During the immature stage, a strong antioxidant activity was observed due to the high presence of polyphenolic compounds; on the other hand, nutritional properties reached the maximum at full ripeness, as well as the caloric intake.

In conclusion, *H. dulcis* has various and useful interesting pharmacological properties and represents a valuable source of active compounds with nutraceutical and pharmaceutical application. However, several issues still need to be explored by basic and clinical research. A better characterization of pharmacological mechanisms of *H. dulcis* effects is needed in order to elucidate the potential medical application of this plant. Furthermore, despite the good tolerability profile demonstrated, an extensive research on pharmacokinetic and safety profile of *H. dulcis* extracts is still lacking. The cases of toxic hepatitis reported in Korea suggest to further investigate the potential toxicological mechanisms in order to better characterize the benefit–risk profile of this plant. *H. dulcis* is of course a promising source of bioactive compounds and a perfect candidate as a food supplement, but the final aim of this review is to underline, once again, that “natural extract” does not mean harmless and misuse has to be avoided. Data shown in this review could be the scientific basis in order to provide knowledge for future studies with the aim to extend the commercialization of the *H. dulcis* extract-based products also into Western countries.

## Figures and Tables

**Table 1:** Compound from *Hovenia Dulcis* isolated from 2005 to now.

Compounds	Chemical Class	Plant Materials	References
(−) Catechine	Flavanol	Stem bark	[9]
2,3,4 Trihydroxybenzoic acid	Organic acid		
(+) Afzelechin	Flavanol		
Caffeine	Xantine	Leaves	[11]
Kaempferol 3,7-*O*-α-l-dirhamnopyranoside	Flavonol triglycoside		
Kaempferol 3-*O*-α-l-rhamno-pyranosyl(1→6-*O*-β-d-glucopyranosyl (1→2)-*O*-β-d-glucopyranoside			
3-*O*-α-l-rhamnopyranoside-7-*O*-[α-d-glucopyranosyl(1→3)-α-l-rhamnopyranoside			
Quercetin 3-*O*-α-l-rhamnopyranoside			
Quercetin 3-*O*-β-d-glucopyranoside			
E-3-carboxy-2-petenedioate 5-methyl ester			
27-*O*-protocatechuoyl-3-dehydroxyisoceanothanolic acid	Ceanothane-type triterpene esters	Roots	[12]
27-*O*-protocatechuoyl-3-dehydroxycolubrinic acid			
27-*O*-protocatechuoyl-3-dehydroxyepicolubrinic acid			
27-*O*-protocatechuoylbetulinic acid	Lupan-type Triterpene esters		
27-*O*-p-hydroxybenzoylbetulinic acid			
27-*O*-syringoylbetulinic acid			
2-methoxybenzoic acid- 5-*O*-α-l-rhamnopyranoside.	Organic acid	Branches	[4]

**Table 2:** Summary of *H. dulcis* pharmacological activities.

Pharmacological Activity **	Active extract	Compounds/Fraction Supposed to be Responsible	Positive Control	Effect/Proposed Mechanism	Type of Evidence	Reference
Alcohol detoxification effect	Water and ethanol fruit, semen and stem bark extracts	Hovenodulinol	n.a.	Reducing alcohol and aldehyde concentration in blood, saliva exhaled breath through the increase of ADH, ALDH and GST activity	In vitro, in vivo and clinical	[8,33,34,35,36,37]
Anti-hangover effect	Water fruit extract	n.a.	n.a.	Changes of IL-6, IL-10, IL-10/IL-6 ratio in serum	Clinical	[38]
Hepatoprotective effect on CCl_4_ liver injury	Methanol fruit extract	DHM	Glycyrrizhin and silymarin	Reduction of AST, ALT and mRNA expression of TIMP-1	In vitro and in vivo	[14,39,40]
Hepatoprotective effect on alcohol induced liver injury	Ethanol fruit and water semen seu fructus extracts	n.a.	Sylimarin	Modulate GSH, SOD, CAT and Nrf2 activities regulation of markers involved in lipogenic (SREBP-1c, SCD1, ACC1, FAS, PPARγ, DGAT2) and fatty acid oxidation (PPARα, ACO1, CPT1) process in liver (CRP, TNF-α and IL-6)	In vivo	[41]
Anticancer effect	Hydro-methanolic and ethanol fruit extracts	n.a.	n.a.	Cytotoxicity against in vitro against different cancer cell lines	In vitro	[42,43]
	Branches ethanol extract	DHM	n.a.	Anti-angiogenic activity inhibiting HIF-1α, VEGFR2 and downstream signaling: STAT3, PKB or AKT and ERK1/2, MMP-2, MMP-9 and cyclin D1	In vitro and in vivo	[21]
Anti-allergic activity	Semen seu fructus methanol extract	Hovenidulciosides A1, A2, B1 B2	n.a.	Inhibitory activity on the histamine release from rat mast cells	In vitro	[44,45,46]
	Ethanol branches extract	Taxifolin, dihydro-kaempferol and pinosylvin	Cetirizine	Inhibition of β-hexosaminidase and histamine release; suppress the FcεRI pathway and inhibits ERK and p38 MAPK. Inhibition of IL-4, TNF-α, PGE2, COX-2, IL-4, NFkB	In vitro and in vivo	[47]
Anti-inflammatory effect	Semen seu fructus ethanol extract	DHM, taxifolin, and myricetin	Dexamethasone	Inhibition of MAPK, AP-1, JAK2/STAT, NF-κB. Reducing the expression of NO and iNOS, TNF-α, IL-6 and IL-1β	In vitro	[48]
	Branches extract	Methyl vanillate	n.a.	Inhibition of TNF-α, IL-6, TARC, MDC, ERK, JNK and p38. Reducing serum IgE and IgG2a levels and the expression of mRNA for Th1- and Th2-related mediators	In vivo	[49]
Laxative effect	Branches water extract	Vanillic acid	n.a.	Improving the intestinal transit and the frequency and weight of stools	In vivo	[50]
Anti-microbial activity	*H. dulcis* water extract	Vanillic and ferulic acids	n.a.	Antimicrobial activity against Gram-positive bacteria, Gram-negative bacteria and yeast	In vitro	[51]
	Pseudo-fruits extract	Catechin and quercetin derivatives	n.a.	Antimicrobial activity against *S. epidermidis*, *S. aureus* and *P. aeruginosa*	In vitro	[3]
Antidiabetic effect	Ethanol seeds extract	Total flavonoids, myricetin and polysaccharides	n.a.	Inhibitory effect of crude extract and its components against α-amylase and α-glucosidase	In vitro	[52]
	Peduncles water extract	n.a.	n.a.	Reduces blood glucose concentration and partially recovers pancreatic islets and pancreatic beta cells	In vivo	[53]
Anti-dyslipidemic and anti-adipogenic activities	Fruit water extract	n.a.	n.a.	Downregulates PPARγ and increases the phosphorylation of AMPK-α	In vitro	[54]
	Hydroalcoholic fruit extract	DHM	n.a.	Reducing total cholesterol and LDL-C in hypercholesterolemic rats	In vivo	[55]
Antioxidant effect	Hydromethanolic pseudo-fruits extracts	Phenolic ompounds	n.a.	Scavenging activity and inhibition of lipid peroxidation process	In vitro	[3]
	Hot water peduncles extract	Polyphenolic–protein–polysaccharide complexes	n.a.	Demonstrated through ABTS, DPPH, NO radical scavenging activities and FRAP methods	In vitro	[56,57]
Anti-osteoporotic effect	Fruits water extract	Methil vanillate	n.a.	Activation of Wnt/β-catenin pathway in in vivo model; increase of mRNA levels of RUNX2, BMP2, ALP and OCN. Increase the expression of RANKL and decrease OPG	In vivo	[58]
Immunomodulatory activity	Aqueous-ethanol peduncle extract	Polysaccharides fraction	n.a.	Stimulating the proliferation of splenocytes and activating peritoneal macrophages enhancing phagocytosis, NO production and acid phosphatase activity	In vitro	[10]
Neuroprotective	Methanol stem bark extract	(−)-catechin and (+)-afzelechin	n.a.	Neuroprotective effect against glutamate-induced neurotoxicity	In vitro	[9]

n.a—no data available; ADH—alcohol dehydrogenase; ALDH—aldehyde dehydrogenase; GST—glutathione S—transferase; IL—interleukin; CCl4—carbon tetrachloride; AST—aspartate aminotransferase; ALT—alanine aminotransferase; TIMP-1—tissue inhibitor matrix metalloproteinase-1; GSH—glutathione; SOD—superoxide dismutase; CAT—catalase; Nrf2—nuclear factor erythroid 2-related factor; SREBP-1c—sterol regulatory element-binding protein 1c; SCD1—stearoyl-CoA desaturase-1; ACC1—acetyl-Coenzyme A carboxylase 1; FAS—fatty acid synthase; PPAR—peroxisome proliferator-activated receptor; DGAT2—diacylglycerol O-acyltransferase; ACO1—aminocyclopropane-1-carboxylic acid oxidase; CPT1—carnitine palmitoyltransferase I; CRP—c-reactive protein; TNF-α—tumor necrosis factor-alfa; HIF-1α—hypoxia-inducible factor 1-alpha; VEGFR2—vascular-endothelial growth factor receptor-2; STAT3—signal transducer and activator of transcription 3; PKB or AKT—protein kinase B; ERK1/2—extracellular signal regulated kinase-1/2; MMP—matrix metalloproteinase; FcεRI—high-affinity immunoglobulin E receptor; p38 MAPK—mammalian p38 mitogen-activated protein kinase,; PGE2—prostaglandin E2; COX-2—cyclooxygenase-2; NFkB—nuclear factor kappa-light-chain-enhancer of activated B cells; AP-1—activator protein-1; JAK2/STAT—janus kinase-2/signal transducer and activator of transcription; NO—nitric oxide; iNOS—nitric oxide synthase; TARC—thymus and activation-regulated chemokine; MDC—macrophage-derived chemokine; JNK—c-jun N-terminal kinase; Ig—immunoglobulin; Th1—type 1 T helper; Th2—type 2 T helper; AMPK-α—activated protein kinase-α; LDL-c—low-density lipoprotein-C; ABTS—2,2′-azino-bis(3-ethylbenzothiazoline-6-sulfonic acid); DPPH—2-2-diphenyl-1-picrylhdrazyl; FRAP—ferric ion reducing antioxidant power; Wnt—wingless-related integration site; RUNX2—runt-related transcription factor 2; BMP2—bone morphogenetic protein 2; ALP—alkaline phosphatase; OCN—osteocalcin; RANKL—receptor activator of nuclear κ B ligand; OPG—osteoprotegerin. * no data available on the type of material used. ** Other scientific data are described in the text.

## Data Availability

No new data were created or analyzed in this study. Data sharing is not applicable to this article.

## References

[1] Hyun T.K., Eom S.H., Yu C.Y., Roitsch T. (2010). *Hovenia dulcis*—An Asian traditional herb. Planta Med..

[2] Tomczyk M., Zovko-Končić M., Chrostek L. (2012). Phytotherapy of alcoholism. Nat. Prod. Commun..

[3] Morales P., Maieves H.A., Dias M.I., Calhella R.C., Sánchez-Mata M.C., Santos-Buelga C., Barros L., Ferreira I.C.F.R. (2017). *Hovenia dulcis* Thunb. pseudofruits as functional foods: Phytochemicals and bioactive properties in different maturity stages. J. Funct. Foods.

[4] Le T.C., Kang K.Y., Yang I., Leutou A.S., Ko J., Son Y.J., Yee S.T., Nam S.J. (2018). A new secondary metabolite from Korean traditional herb plant *Hovenia dulcis*. Nat. Prod. Commun..

[5] Xu B.J., Deng Y.Q., Lee J.H., Mo E.K., Sung C.K. (2003). Chemical compositions of the genus *Hovenia*. Nat. Prod. Sci..

[6] Wang M., Zhu P., Jiang C., Ma L., Zhang Z., Zeng X. (2012). Preliminary characterization, antioxidant activity in vitro and hepatoprotective effect on acute alcohol-induced liver injury in mice of polysaccharides from the peduncles of *Hovenia dulcis*. Food Chem. Toxicol..

[7] Takai M., Ohihara Y., Shibata S. (1973). New peptide alkaloids from *Hovenia dulcis* and *H. tomentella*. Phytochemistry.

[8] Hovenodulinol, An Active Compound Extracted from *Hovenia dulcis* Thunb., A Process for Preparing the Same, And an Alcohol Decomposing Agent or an Agent for Alleviating Lingering Intoxication Containing the Same. https://patents.google.com/patent/WO2002024678A1/en.

[9] Li G., Min B.S., Zheng C., Lee J., Oh S.R., Ahn K.S., Lee H.K. (2005). Neuroprotective and Free Radical Scavenging Activities of Phenolic Compounds from *Hovenia dulcis*. Arch. Pharm. Res..

[10] Wang M., Jiang C., Ma L., Zhang Z., Cao L., Liu J., Zeng X. (2013). Preparation, preliminary characterization and immunostimulatory activity of polysaccharide fractions from the peduncles of *Hovenia dulcis*. Food Chem..

[11] Cho J.Y., Hyun S.H., Moon J.K., Park K.H. (2013). Isolation and Structural Determination of a Novel Flavonol Triglycoside and 7 Compounds from the leaves of oriental raisin tree (*Hovenia dulcis*) and their antioxidative activity. Food Sci. Biotechnol..

[12] Kang K.B., Jun J.B., Kim J.W., Kim H.W., Sung S.H. (2017). Ceanothane-and lupane-type triterpene esters from the roots of *Hovenia dulcis* and their antiproliferative activity on HSC-T6 cells. Phytochemistry.

[13] Linsheng D., Qiaoli L., Yanfen T. (1997). Study on flavonoids in seeds of *Hovenia dulcis*. Yao Xue Xue Bao.

[14] Hase K., Ohsugi M., Xiong Q., Basnet P., Kadota S., Namba T. (1997). Hepatoprotective effect of *Hovenia dulcis* Thunb. on experimental liver injuries induced by carbon tetrachloride or d-galactosamine/lipopolysaccharide. Biol. Pharm. Bull..

[15] Kotake M., Kubota T. (1940). Constituents of *Ampelopsis meliaefolia* Kudo (Haku-tya). Justus Liebigs Ann. Chem..

[16] Zhou T., Zhou X. (1996). Isolation, structure determination and pharmacological activity of a flavanonol from *Ampelopsis grossedentata*. Zhongguo Yaoxue Zazhi.

[17] Liu D.Y., Lei H.Q. (1996). Inhibitory Effects of Myricetin and Ampelopsin on Tyrosinase. Shengwu Huaxue Zazhi.

[18] Yoshikawa M., Murakami T., Ueda T., Yoshizumi S., Ninomiya K., Murakami N., Matsuda H., Saito M., Fujii W. (1997). Bioactive constituents of Chinese natural medicines. III. Absolute stereostructures of new dihydroflavonols, hovenitins I, II, and III, isolated from *Hoveniae* Semen Seu Fructus, the seed and fruit of *Hovenia dulcis* Thunb. (Rhamnaceae): Inhibitory effect on alcohol-induced muscular relaxation and hepatoprotective activity. Yakugaku Zasshi.

[19] Shen Y., Lindemeyer A.K., Gonzalez C., Shao X.M., Spigelman I., Olsen R.W., Liang J. (2012). Dihydromyricetin as a novel anti-alcohol intoxication medication. J. Neurosci..

[20] Zhou Y., Mao T.Y. (2014). Influence of dihydromyricetin on lowering blood glucose concentration and reducing early kidney damage in imparied glucose tolerance rats. Adv. Mater. Res..

[21] Mi H.J., Nim L.H., Jin J.H. (2017). *Hovenia dulcis* Thunb. and its active compound ampelopsin inhibit angiogenesis through suppression of VEGFR2 signaling and HIF-1α expression. Oncol. Rep..

[22] Dong S., Ji J., Zhang B., Hu L., Cui X., Wang H. (2018). Protective effects and possible molecular mechanism of *Hovenia dulcis* Thunb. extract on acetaminophen-induced hepatotoxicity. Pharmazie.

[23] Sijing D., Jianbo J., Lingyun H., Haina W. (2019). Dihydromyricetin alleviates acetaminophen-induced liver injury via the T regulation of transformation, lipid homeostasis, cell death and regeneration. Life Sci..

[24] Liu D., Mao Y., Ding L., Zeng X.A. (2019). Dihydromyricetin: A review on identification and quantification methods, biological activities, chemical stability, metabolism and approaches to enhance its bioavailability. Trends Food Sci. Technol..

[25] Liu S.S., Dhanwani S.B., Kumar R. (2012). Microemulsion drug delivery system: For bioavailability enhancement of ampelopsin. Pharmaceutics.

[26] Chenguang W., Qing T., Xiaolong H., Shenye H., Jianguo F., Calvin C.S. (2016). Enhancing Bioavailability of Dihydromyricetin through Inhibiting Precipitation of Soluble Cocrystals by a Crystallization Inhibitor. Cryst. Growth Des..

[27] Dalcin A.J.F., Santos C.G., Gundel S.S., Roggia I., Raffin R.P., Ourique A.F., Santos R.C.V., Gomes P. (2017). Anti biofilm effect of dihydromyricetin-loaded nanocapsules on urinary catheter infected by *Pseudomonas aeruginosa*. Colloids Surf. B Biointerfaces.

[28] Hongwei Z., Qingrong H. Hydrophilic modification of zein protein and its application in amorphous solid dispersions to enhance dissolution of Dihydromyricetin. Proceedings of the 255th ACS National Meeting & Exposition.

[29] Dantong W., Yudi M., Qiang W., Juan H., Rui S., Qiang X. (2019). Solid Self-Emulsifying delivery system (S-SEDS) of dihydromyricetin: A new way for preparing functional food. J. Food Sci..

[30] Xinyuan Z., Chunyang S., Xiya Z., Tong L., Yusheng G., Mingxing T., Li F., Wenqing W., Jianguo F. (2019). Preparation of a nanoscale dihydromyricetin-phospholipid complex to improve the bioavailability: In vitro and in vivo evaluations. Eur. J. Pharm. Sci..

[31] Chen L., Lin X., Teng H. (2020). Emulsions loaded with dihydromyricetin enhance its transport through Caco-2 monolayer and improve anti-diabetic effect in insulin resistant HepG2 cell. J. Funct. Foods..

[32] Li H., Li Q., Liu Z., Yang K., Chen Z., Cheng Q., Wu L. (2017). The Versatile Effects of Dihydromyricetin in Health. Evid. Based Complement. Alternat. Med..

[33] Xu B.J., Deng Y.Q., Jia X.Q., Lee J.H., Mo E.K., Kang H.J., Sung C.K. (2003). A rapid screening for alcohol detoxification constituents of *Hovenia dulcis* by microplate reader. Agric. Chem. Biotechnol..

[34] Kim K.H., Chung Y.T., Lee J.H., Park Y.S., Shin M.K., Kim H.S., Kim D.H., Lee H.Y. (2000). Hepatic detoxification activity and reduction of serum alcohol concentration of *Hovenia dulcis* Thunb. from Korea and China. Korean J. Med. Crop Sci..

[35] Okuma Y., Ishikawa H., Ito Y., Hayashi Y., Endo A., Watanabe T. (1995). Effect of extracts from *Hovenia dulcis* Thunb. on alcohol concentration in rats and men administered alcohol. Jpn. Nutr. Crop. Sci. Bull..

[36] Chen S.H., Zhong G.S., Li A.L., Li S.H., Wu L.K. (2006). Influence of *Hovenia dulcis* on alcohol concentration in blood and activity of alcohol dehydrogenase (ADH) of animals after drinking. Zhongguo Zhong Yao Za Zhi.

[37] Du J., He D., Sun L.N., Han T., Zhang H., Qin L.P., Rahman K. (2010). Semen Hoveniae extract protects against acute alcohol-induced liver injury in mice. Pharm. Biol..

[38] Kim H., Kim Y.J., Jeong H.Y., Kim J.Y., Choi E.K., Chae S.W., Kwon O. (2017). A standardized extract of the fruit of *Hovenia dulcis* alleviated alcohol-induced hangover in healthy subjects with heterozygous ALDH2: A randomized, controlled, crossover trial. J. Ethnopharmacol..

[39] Liu X.L., Zhnag H., Wang F. (2006). Effect of *Hovenia dulcis* Extract on Expression of MMP-13 and TIMP-1 in Hepatic Tissue. Zhongguo Zhong Yao Za Zhi.

[40] Grünwald B., Schoeps B., Krüger A. (2019). Recognizing the Molecular Multifunctionality and Interactome of TIMP-1. Trends Cell Biol..

[41] Cho I., Kim J., Jung J., Sung S., Kim J., Lee N., Ku S. (2016). Hepatoprotective effects of hoveniae semen cum fructus extracts in ethanol intoxicated mice. J. Exerc. Nutr. Biochem..

[42] Castro T.C., Pelliccione V.L.B., Figueiredo M.R., Soares R.O.A., Bozza M.T., Viana V.R.C., Albarello N., Solange F.L.F. (2002). Atividade antineoplásica e tripanocida de *Hovenia dulcis* Thunb. cultivada in vivo e in vitro. Rev. Bras. Farmacogn..

[43] Park S.H., Chang E.Y. (2007). Antimutagenic and cytotoxic effects of *Hovenia dulcis* Thumb. leaves extracts. Korean J. Soc. Food. Sci. Nutr..

[44] Xu B.J., Deng Y.Q., Sung C.K. (2004). Advances in Studies on Bioactivity of *Hovenia dulcis*. Agric. Chem. Biotechnol..

[45] Yoshikawa M., Ueda T., Muraoka O., Aoyama H., Matsuda H., Shimoda H., Yamahara J., Murakami N. (1995). Absolute stereostructures of hovenidulciosides A1 and A2, bioactive novel triterpene glycosides from Hoveniae semen seu fructus, the seeds and fruit of *Hovenia dulcis* Thunb. Chem. Pharm. Bull..

[46] Yoshikawa M., Murakami T., Ueda T., Matsuda H., Yamahara J., Murakami N. (1996). Bioactive saponins and glycosides. IV. Four methyl-migrated 16, 17-seco-damma-rane triterpene gylcosides from Chinese natural medicine, Hoveniae semen seu fructus, the seeds and fruit of *Hovenia dulcis* Thunb.: Absolute stereostructures and inhibitory activity on histamine release of hovenidulciosides A1, A2, B1, and B2. Chem. Pharm. Bull..

[47] Lim S.J., Kim M., Randy A., Nho C.W. (2015). Inhibitory effect of the branches of *Hovenia dulcis* Thunb. and its constituent pinosylvin on the activities of IgE-mediated mast cells and passive cutaneous anaphylaxis in mice. Food Funct..

[48] Jeong Y.H., Oh Y.C., Cho W.K., Yim N.H., Ma J.Y. (2019). Hoveniae Semen Seu Fructus Ethanol Extract Exhibits Anti-Inflammatory Activity via MAPK, AP-1, and STAT Signaling Pathways in LPS-Stimulated RAW 264.7 and Mouse Peritoneal Macrophages. Mediat. Inflamm..

[49] Lim S.J., Kim M., Randy A., Nam E.J., Nho C.W. (2016). Effects of *Hovenia dulcis* Thunb. extract and methylvanillate on atopic dermatitis-like skin lesions and TNF-a/IFN-c-induced chemokines production in HaCaT cells. J. Pharm. Pharmacol..

[50] Oh K.N., Kim Y., Choi E.J., Lee H., Hong J.A., Kim M., Oh D.R., Jung M.A., Park R.D., Kim S.I. (2020). Laxative activity of the hot-water extract mixture of *Hovenia dulcis* Thunb. and *Phyllostachys pubescens* Mazel in chronic constipation model SD rats. J. Microbiol. Biotechnol..

[51] Cho J.Y., Moon J.H., Park K.H. (2000). Isolation and identification of 3-methoxy-4-hydroxybenzoic acid and 3-methoxy-4hydroxycinnamic acid from hot water extracts of *Hovenia dulcis* Thunb and confirmation of their antioxidative and antimicrobial activity. Korean J. Food. Sci. Technol..

[52] Meng Y., Su A., Yuan S., Zhao H., Tan S., Hu C., Deng H., Guo Y. (2016). Evaluation of total flavonoids, myricetin, and 1uercetin from *Hovenia dulcis* Thunb. as inhibitors of α-amylase and α-glucosidase. Plant Foods Hum. Nutr..

[53] Kim J.S., Na C.S., Eun B.J. (2005). Effect of *Hovenia dulcis* Thunber extract on the hyperglycemic mice induced with streptozotocin. J. Korean Soc. Food Sci. Nutr..

[54] Kim H.L., Sim J.E., Choi H.M., Choi I.Y., Jeong M.Y., Park J., Jung Y., Youn D.H., Cho J.H., Kim J.H. (2014). The AMPK pathway mediates an anti-adipogenic effect of fruits of *Hovenia dulcis* Thunb. Food Funct..

[55] Pinto J.T., Toledo de Oliveira T., Alvarenga L.F., Barbosa A.S., Ramos Pizziolo V., Da Costa M.R. (2014). Pharmacological activity of the hydroalcoholic extract from *Hovenia dulcis* thunberg fruit and the flavonoid dihydromyricetin during hypercholesterolemia induced in rats. Braz. J. Pharm. Sci..

[56] Yang B., Luo Y., Wu Q., Yang Q., Kan J. (2020). *Hovenia dulcis* polysaccharides: Influence of multi-frequency ultrasonic extraction on structure, functional properties, and biological activities. Int. J. Biol. Macromol..

[57] Liu W., Li F., Wang P., Liu X., He J.J., Xian M.L., Zhao L., Qin W., Gan R.Y., Wu D.T. (2020). Effects of drying methods on the physicochemical characteristics and bioactivities of polyphenolic-protein-polysaccharide conjugates from *Hovenia dulcis*. Int. J. Biol. Macromol..

[58] Cha P.H., Shin W., Zahoor M., Kim H.Y., Min D.S., Choi K.Y. (2014). *Hovenia dulcis* Thunb extract and its ingredient methyl vanillate activate wnt/ β-catenin pathway and increase bone mass in growing or ovariectomized mice. PLoS ONE.

[59] Kim Y.S., Park J., Kwon Y., Lim D.W., Song M.K., Choi H.Y., Kim H. (2013). Hepatoprotective Effects of *Hovenia dulcis* Extract on Acute and Chronic Liver Injuries induced by Alcohol and Carbon Tetrachloride. Korean J. Herbol..

[60] Lee J.J., Yang D., Kim H., Hur S.J., Lee J.D., Yum M.J., Song M.D. (2014). Liver Fibrosis Protective Effect of *Hovenia dulcis* fruit. Curr. Top. Nutraceutical Res..

[61] Fang H., Lin H.Y., Chan M.C., Lin W.L., Lin W.C. (2007). Treatment of Chronic Liver Injuries in Mice by Oral Administration of Ethanolic Extract of the Fruit of *Hovenia dulcis*. Am. J. Chin. Med..

[62] Na C.S., Jung N.C. Lower Alcohol-Insoluble Extract of *Hovenia dulcis* var. Koreana nakai, A Polysaccharide Isolated Thereform and an Antihepatotoxic Composition Containing Same. https://patents.google.com/patent/WO2002060463A1/en.

[63] Choi R.Y., Woo M.J., Ham J.R., Lee M.K. (2017). Anti-steatotic and anti-inflammatory effects of *Hovenia dulcis* Thunb. extracts in chronic alcohol-fed rats. Biomed. Pharmacother..

[64] Ping Q., Yu D., Tao Z., Yun-yun L., Xian-jie K., Min-xia P., Huan-Zhou L., Hao X., Chao G., Su-hua P. (2019). Semen hoveniae extract ameliorates alcohol-induced chronic liver damage in rats via modulation of the abnormalities of gut-liver axis. Phytomedicine.

[65] Bui N. (2019). Hepatoprotective Activity of *Hovenia dulcis* Thumb. on paracetamol Induced Liver Toxicity in Mice. Gut Liver.

[66] Hase K., Ohsugi M., Basnet P., Kadota S., Namba T. (1997). Effect of *Hovenia dulcis* on lipopolysaceharide-induced liver injury in chronic alcohol-fed rats. J. Trad. Med..

[67] Park J.Y., Moon J.Y., Park S.D., Park W.H., Kim H., Kim J.E. (2016). Fruits extracts of *Hovenia dulcis* Thunb. Suppresses lipopolysaccharide-stimulated inflammatory responses through nuclear factor-kappa B pathway in Raw 264.7 cells. Asian Pac. J. Trop. Med..

[68] Lee J.S., Lee M.K., Kim Y.K., Kim K.E., Hyun K.Y. (2014). Attenuant effects of *Hovenia dulcis* extract on inflammatory orofacial pain in rats. J. Korea Acad. Ind. Coop. Soc..

[69] Choi C.Y., Cho S.S., Yoon I.S. (2018). Hot-water extract of the branches of *Hovenia dulcis* Thunb. (Rhamnaceae) ameliorates low-fiber diet-induced constipation in rats. Drug Des. Devel. Ther..

[70] Cho J.Y., Moon J.H., Eun J.B., Chung S.J., Park K.H. (2006). Isolation and characterization of 3(Z)-dodecenedioic acid as an antibacterial substance from *Hovenia dulcis* Thunb. Food Sci. Biotechnol..

[71] Basavegowda N., Idhayadhulla A., Lee Y.R. (2014). Phyto-synthesis of gold nanoparticles using fruit extract of *Hovenia dulcis* and their biological activities. Ind. Crops Prod..

[72] Gadelha A.P.R., Vidal F., Castro T.M., Lopes C.S., Albarello N., Coelho M.G.P., Figueiredo S.F.L., Leal-Monteiro L.H. (2005). Susceptibility of Giardia lamblia to *Hovenia dulcis* extracts. Parasitol. Res..

[73] Wu D.T., Liu W., Xian M.L., Du G., Liu X., He J.J., Wang P., Qin W., Zhao L. (2020). Polyphenolic-protein-polysaccharide complexes from *Hovenia dulcis*: Insights into extraction methods on their physicochemical properties and in vitro bioactivities. Foods.

[74] Yang B., Wu Q., Song X., Yang Q., Kan J. (2019). Physicochemical properties and bioactive function of Japanese grape (*Hovenia dulcis*) pomace insoluble dietary fiber modified by ball milling and complex enzyme treatment. Int. J. Food Sci. Technol..

[75] Yang B., Wu Q., Luo Y., Yang Q., Chen G., Wei X., Kan J. (2019). Japanese grape (*Hovenia dulcis*) polysaccharides: New insight into extraction, characterization, rheological properties, and bioactivities. Int. J. Biomacromol..

[76] Ji Y., Chen S., Zhang K., Wan W. (2002). Effects of *Hovenia dulcis* Thunb on blood sugar and hepatic glycogen in diabetic mice. Zhong Yao Cai.

[77] Lee Y.A., Chae H.J., Moon H.Y. (2005). Effect of *Hovenia dulcis* Thunb. var. koreana Nakai fruit extract on glucose, lipid metabolism and antioxidant activity in streptozotocin-induced diabetic rats. J. Exp. Biomed. Sci..

[78] Wu L., Zhang J. (2012). Evaluation of anti-diabetic activities of *Hovenia dulcis* Thunb. Adv. Mater. Res..

[79] De Godoi R.S., Almera M.P., da Silva F.R. (2020). In silico evaluation of the antidiabetic activity of natural compounds from *Hovenia dulcis* Thunberg. J. Herbal Med..

[80] Cho I.J., Kim J.W., Jung J.J., Sung S.H., Ku S.K., Choi J.S. (2016). In Vitro Protective Effects of Hoveniae Semen cum Fructus Extracts against Oxidative Stress. Toxicol. Environ. Health Sci..

[81] Liu Y., Qiang M., Sun Z., Du Y. (2015). Optimization of ultrasonic extraction of polysaccharides from *Hovenia dulcis* peduncles and their antioxidant potential. Int. J. Biol. Macromol..

[82] Yang X., Yan L., Liu T., Zhang Q., Zhao Y., Yu M., Yu Z. (2019). Simultaneous determination of bioactive flavonoids of Hoveniae Semen in rat plasma by LC-MS/MS: Application to a comparative pharmacokinetic study. J. Cromatogr. B.

[83] Park J.S., Rehman S.U., Kim I.S., Choi M.S., Na C.S., Yoo H.H. (2017). Evaluation of herb–drug interactions of *Hovenia dulcis* fruit extracts. Pharmacogn. Mag..

[84] Fan L., Tong Q., Dong W., Yang G., Hou X., Xiong W., Wang W. (2017). Tissue distribution, excretion, and metabolic profile of dihydromyricetin, a flavonoid from vine tea (*Ampelopsis grossedentata*) after oral administration in rats. J. Agric. Food Chem..

[85] Tong Q., Hou X., Fang J., Wang W., Xiong W., Liu X., Shi C. (2015). Determination of dihydromyricetin in rat plasma by LC-MS/MS and its application to a pharmacokinetic study. J. Pharm. Biomed. Anal..

[86] Huang Y., Xu Z.S., Ye Q. (2013). Effect of dihydromyricetin on cytochrome P450 isoforms CYP1A2, CYP2C9 and CYP3A4 in rats. Lat. Am. J. Pharm..

[87] Çelik H., Koşar M., Arinç E. (2013). In vitro effects of myricetin, morin, apigenin, (+)-taxifolin, (+)-catechin, (−)-epicatechin, naringenin and naringin on cytochrome b5 reduction by purified NADH-cytochrome b5 reductase. Toxicology.

[88] Maieves H.A., Ribani R.H., Morales P., Sánchez-Mata M.C. (2015). Evolution of the nutritional composition of *Hovenia dulcis* Thunb. pseudofruit during the maturation process. Fruits.

[89] Liu Y., Cai B., Qiang M. (2017). Characterization and bioactivities of polysaccharide from spent *Hovenia dulcis* peduncles by alkali pretreatment. Int. J. Food Prop..

[90] De Biaggi M., Donno D., Mellano M.G., Gamba G., Riondato I., Rakotoniaina E.N., Beccaro L.B. (2020). Emerging species with nutraceutical properties: Bioactive compounds from *Hovenia dulcis* pseudofruits. Food Chem..

[91] Hu W., Lee K., Wang M.H. (2010). Antioxidant Activities of Various Extracts of *Hovenia dulcis* Thunb. Fruits. Korean J. Plant Res..

[92] Kim B., Woo M.J., Park C.S., Lee S.H., Kim J.S., Kim B., An S., Kim S.H. (2017). *Hovenia dulcis* extract reduces lipid accumulation in oleic acid-induced steatosis of Hep G2 cells via activation of AMPK and PPARα/CPT-1 pathway and in acute hyperlipidemia mouse model. Phytother. Res..

[93] Teschke R., Eickhoff A. (2015). Herbal hepatotoxicity in traditional and modern medicine: Actual key issues and new encouraging steps. Front. Pharmacol..

[94] Kim Y.J., Ryu S.L., Shim J.W., Kim D.S., Shim J.Y., Park M.S., Jung H.L. (2012). A pediatric case of toxic hepatitis induced by *Hovenia Dulcis*. Pediatr. Gastroenterol. Hepatol. Nutr..

[95] Kang S.H., Kim J.I., Jeong K.H., Ko K.H., Ko P.G., Hwang S.W., Kim E.M., Kim S.H., Lee H.Y., Lee B.S. (2008). Clinical characteristics of 159 cases of acute toxic hepatitis. Korean J. Hepatol..

[96] Sohn C.H., Cha M.I., Oh B.J., Yeo W.H., Lee J.H., Kim W., Lim K.S. (2008). Liver transplantation for acute toxic hepatitis due to herbal medicines and preparations. J. Korean Soc. Clin. Toxicol..

[97] Bernardi F., Possa M.G., Faccin M., Gruchouskei L., Fonseca-Alves C.E., Pípole F., Retzde Carvalho L., Elia F. (2016). Spontaneous poisoning by *Hovenia dulcis* in dairy cattle in Southwest Parana, Brazil. Trop. Anim. Health Prod..

[98] Cardoso T.C., Emmerich T., Wicpolt N.S., Ogliari D., Traverso S.D., Gava A. (2015). Experimental poisoning by the fruits of *Hovenia dulcis* (Rhamnaceae) in cattle. Pesq. Veterin. Bras..

[99] Turck D., Castenmiller J., De Henauw S., Hirsch-Hernst K.I., Kearney J., Maciuk A., Mangelsdorf I., McArdle H.J., Naska A., EFSA Panel on Nutrition, Novel Foods and Food Allergens (NDA) (2020). Safety of hot water extract of fruits and peduncles of *Hovenia dulcis* as a novel food pursuant to Regulation 1(EU) 2015/2283. EFSA J..

[100] European Commission Novel Food. https://ec.europa.eu/food/safety/novel_food_en.

[101] Turck D., Bresson J.L., Burlingame B., Dean T., Fairweather-Tait S., Heinonen M., Hirsch-Ernst K.I., Mangelsdorf I., McArdle H., EFSA Panel on Nutrition, Novel Foods and Food Allergens (NDA) (2016). Guidance on the preparation and presentation of an application for authorisation of a novel food in the context of Regulation (EU) 2015/2283. EFSA J..

[102] Turck D., Bresson J.L., Burlingame B., Dean T., Fairweather-Tait S., Heinonen M., Hirsch-Ernst K.I., Mangelsdorf I., McArdle H., EFSA Panel on Nutrition, Novel Foods and Food Allergens (NDA) (2016). Guidance on the preparation and presentation of the notification and application for authorisation of traditional foods from third countries in the context of Regulation (EU) 2015/2283. EFSA J..

[103] European Commission Summary of the Applications Submitted within the Meaning of Article 10 (1) of Regulation (EU) 2105/2283. https://ec.europa.eu/food/safety/novel_food/authorisations/summary-applications-and-notifications_en).

[104] FSA Advisory Committee on Novel Foods and Processes Advisory Committee for Novel Foods and Processes—*Hovenia dulcis* Fruit Extract—Dossier 163. https://acnfp.food.gov.uk/sites/default/files/acnfp-132-07hoveniadulcis.pdf.

